# Dynamic Covalent Amphiphilic Polymer Conetworks Based on End-Linked Pluronic F108: Preparation, Characterization, and Evaluation as Matrices for Gel Polymer Electrolytes

**DOI:** 10.1021/acsami.3c19189

**Published:** 2024-04-26

**Authors:** Demetris E. Apostolides, George Michael, Costas S. Patrickios, Benoît Notredame, Yinghui Zhang, Jean-François Gohy, Sylvain Prévost, Michael Gradzielski, Florian A. Jung, Christine M. Papadakis

**Affiliations:** 1 Department of Chemistry, University of Cyprus, P.O. Box 20537, 1678 Nicosia, Cyprus; 2 Institute for Condensed Matter and Nanosciences (IMCN), Bio- and Soft Matter (BSMA), Université Catholique de Louvain (UCL), Place Pasteur 1, 1348 Louvain-la-Neuve, Belgium; 3 Institut Max von Laue—Paul Langevin (ILL), 71, Avenue des Martyrs—CS 20156, 38042 Grenoble Cedex 9, France; 4 Stranski-Laboratorium für Physikalische und Theoretische Chemie, Institut für Chemie, Technische Universität, Straße des 17, Juni 124, D-10623 Berlin, Germany; 5 Soft Matter Physics Group, Physics Department, TUM School of Natural Sciences, Technical University of Munich, James-Franck-Straße 1, 85748 Garching, Germany

**Keywords:** amphiphilic polymer conetworks, self-assembly, stretchability, small-angle neutron scattering, Pluronics, gel polymer electrolytes, ion conductivity

## Abstract

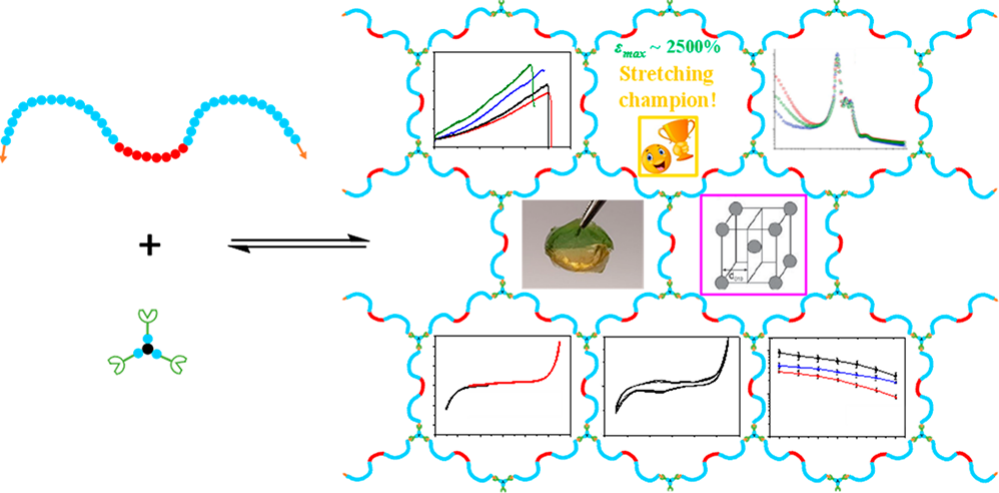

We present the development of a platform of well-defined, dynamic covalent amphiphilic polymer conetworks (APCN) based on an α,ω-dibenzaldehyde end-functionalized linear amphiphilic poly(ethylene glycol)-*b*-poly(propylene glycol)-*b*-poly(ethylene glycol) (PEG-*b*-PPG-*b*-PEG, Pluronic) copolymer end-linked with a triacylhydrazide oligo(ethylene glycol) triarmed star cross-linker. The developed APCNs were characterized in terms of their rheological (increase in the storage modulus by a factor of 2 with increase in temperature from 10 to 50 °C), self-healing, self-assembling, and mechanical properties and evaluated as a matrix for gel polymer electrolytes (GPEs) in both the stretched and unstretched states. Our results show that water-loaded APCNs almost completely self-mend, self-organize at room temperature into a body-centered cubic structure with long-range order exhibiting an aggregation number of around 80, and display an exceptional room temperature stretchability of ∼2400%. Furthermore, ionic liquid-loaded APCNs could serve as gel polymer electrolytes (GPEs), displaying a substantial ion conductivity in the unstretched state, which was gradually reduced upon elongation up to a strain of 4, above which it gradually increased. Finally, it was found that recycled (dissolved and re-formed) ionic liquid-loaded APCNs could be reused as GPEs preserving 50–70% of their original ion conductivity.

## Introduction

Synthetic polymer networks constitute a major portion of the global synthetic polymer production,^1^ representing about one-fourth of that and amounting to an estimated 86 million tons in 2018 with a total value of 247 billion US$ and an average unit price of 2.86 US$ kg^–1^.^2^ From the four classes of synthetic polymer networks, thermosetting polymers (thermosets), elastomers (rubbers), thermoplastic elastomers, and gels,^3^ the last one is the one with the highest average unit price of 7.61 US$ kg^–1^ and with total sales in 2019 of more than 22 billion US$. That amount of sales came approximately equally from the rather inexpensive superabsorbents and the much more expensive silicone hydrogel soft contact lenses,^4^ with the latter high-added-value product justifying the highest average unit price for this type of synthetic polymer networks.

With annual global sales of more than 10 billion US$, silicone hydrogel soft contact lenses possess an intriguing polymer architecture, that of amphiphilic polymer conetworks (APCNs).^5^ APCNs comprise covalently cross-linked hydrophilic and hydrophobic segments. Upon equilibration in an aqueous environment, APCNs self-organize as their hydrophilic and hydrophobic components separate into their respective nanophases. The hydrophobic segments in the above-mentioned type of contact lenses comprise silicone, i.e., cross-linked polydimethylsiloxane, which exhibits high oxygen permeability, facilitating eye oxygenation, necessitated by the fact that the cornea lacks blood vessels. On the other hand, the contact lens is comfortable to the eye because of its softness, due to the fact that both nanophases are soft: silicone is intrinsically soft (glass transition temperature is −130 °C), and the hydrophilic nanophase becomes soft due to its hydration in the ocular milieu.

Although silicone hydrogel soft contact lenses constitute their most successful commercial application,^5,6^ APCNs have some important emerging applications in both biomedicine and technology. Future biomedical uses of APCNs^7^ include their utilization as scaffolds for drug (both hydrophobic and hydrophilic^6^) delivery and tissue engineering, as porous matrices for phase transfer organic and enzymatic catalyses^8^ and as antimicrobial yet cytocompatible membranes.^9^ Offering themselves as materials for the sequestration of oil pollutants,^10^ as matrices for luminescent solar concentrators^11^ and as matrices for solid^12,13^ or gel,^14^ polymer electrolytes constitute some of the promising APCN uses in technology. Further potential APCN applications are their use as highly efficient separation membranes for small molecule racemates^15^ and proteins.^16^

We have an interest in the use of APCNs as matrices in gel or solid polymer electrolytes in lithium ion batteries (LIBs).^17^ Their chemically cross-linked structure confers upon APCN-based polymer electrolytes mechanical durability and dimensional stability, enabling them to also act as separators, in addition to serving as electrolytes in LIBs. However, the presence of the cross-links adversely affects the large-scale manufacturing and postlifespan recycling of these materials. The use of dynamic covalent cross-links, rather than irreversible chemical cross-links, would render APCN-based polymer electrolytes processable and recyclable without compromising their network characteristics. Although there are several examples in the literature on self-healable polymer network electrolytes,^18−20^ there are only a small number of examples also including their recycling, with these examples concerning only networks based on simple building blocks and not APCNs.^21−26^ Thus, the recycling and re-evaluation of APCN-based electrolytes bearing dynamic covalent cross-links are yet to be explored, something undertaken within the present study.

Another objective in this study is to improve the quality of the nanophase separation in APCNs. The presence of cross-links prevents APCNs from self-organizing into morphologies with minimized interfacial areas, resulting in APCN nanophase separating into distorted, usually spheroidal, structures with only short-range order.^6−14,27−36^ However, some relatively recent studies have shown that APCN nanophase separation with long-range order is possible when a minimal amount of cross-linker is used^37^ and when the building blocks are all well-defined.^38^ Furthermore, a very recent simulations investigation has indicated that bulk melts of model APCNs, i.e., APCNs with an ideal structure, microphase-separate into morphologies almost identical to those of their linear and free star counterparts, with the latter two architectures lacking the constraints imposed by cross-links.^39^ Thus, their careful design and preparation from components possessing an almost perfect structure may yield APCNs that upon self-assembly can give morphologies with long-range order.

Dictated by the literature surveyed in the two preceding paragraphs, in the present study we prepared APCNs based on well-defined building blocks^40^ interconnected via dynamic covalent cross-links,^41^ acylhydrazone,^42^ in particular. The resulting APCNs were subsequently characterized in terms of their aqueous self-assembling capability in order to examine if the obtained morphologies possessed long-range order. Afterward, these APCNs were loaded with an ionic liquid, and the resulting polymer network electrolytes were characterized in terms of their electrochemical stability and ion conductivity. Furthermore, the APCN-based polymer network electrolytes were dissolved and re-formed, and the ionic conductivity of the recycled polymer network electrolytes was evaluated and compared to that of the pristine material. Finally, owing to their high extensibility, the presently developed APCN-based polymer network electrolytes stretched up to 10 times their original length were characterized in terms of their ion conductivity, which was compared to that of the same material in the unstretched state.

## Experimental Section

Full details of the Experimental Section are provided in the Supporting Information, whereas a brief account is given below.

### Materials

All materials were purchased from Sigma-Aldrich-Merck, Germany, with the exception of the ionic liquid mixture, which was obtained from Solvionic, France.

### Synthetic Methods

End-functionalization of Pluronic F108 followed our previously developed procedure^14,43^ which was based on a modification of Deng’s original procedure.^44^ Similarly, formation of aqueous APCNs^43^ and APCN-based gel polymer electrolytes^14^ was based on our previously published methods.

### Characterization Methods

Proton nuclear magnetic resonance (^1^H NMR) spectroscopy was performed by using a Bruker 500 MHz Avance spectrometer, whereas rheology was performed on a Discovery HR2 rheometer from Thermal Analysis Instruments. The network mechanical properties were evaluated using an Instron 5944 mechanical tester, while small-angle neutron scattering (SANS) was performed at the Institut Laue-Langevin (ILL) in Grenoble, France. Finally, electrochemical stability and ion conductivity measurements employed a BioLogic VMP3 potentiostat.

## Results and Discussion

### Two APCN Components

Preparation of well-defined APCNs requires the utilization of equally well-defined building blocks. Even better-defined APCN structures may be afforded via the end-linking of these building blocks.^45^ To be end-linkable, the building blocks must be end-functionalized with complementary reactive terminal groups. As building blocks, we chose two commercially available polymers, originally both bearing hydroxyl end groups, which had to be converted to two different functionalities that could react to each other. Since our design dictated self-healable and recyclable APCNs, this end-linking reaction should give a dynamic covalent bond. From the two chosen polymeric components, one was larger and linear, carrying two terminal hydroxyl groups (main building block), and the other was smaller (oligomeric) but branched, carrying three terminal hydroxyl groups (cross-linker).

#### Main Building Block

**Figure 1:**
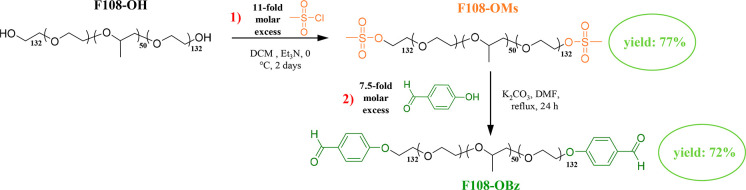
Two-step procedure followed for the attachment of benzaldehyde groups at the F108 termini.

This was a linear amphiphilic ABA triblock copolymer with a poly(propylene glycol) (PPG) midblock and poly(ethylene glycol) (PEG) end-blocks, i.e., a Pluronic copolymer and, in particular, Pluronic F108. F108 has the structure EG_132_-*b*-PG_50_-*b*-EG_132_ and two hydroxyl end-groups. Its molar mass is 14 600 g mol^–1^, and its hydrophobic PG mole fraction is 0.16, corresponding to a 0.20 mass fraction.^46^ Our network design involved the end-linking of the F108 polymeric building block through a water-soluble tri(acylhydrazide) cross-linker (AGE, see below). To enable this, F108 had to be end-functionalized with benzaldehyde groups which react with acylhydrazide groups to form acylhydrazone dynamic covalent bonds.^42^Figure 1 illustrates the end-group modification of Pluronic F108, leading to its α,ω-bisbenzaldehyde derivative in two synthetic steps following our modification^43^ of the procedure developed by Deng et al.^44^ The first step involves the mesylation of the hydroxyl end-groups, followed by their displacement in the second and final step using 4-hydroxybenzaldehyde, at yields of 77 and 72%, respectively.

The ^1^H NMR spectra of the starting α,ω-dihydroxyl Pluronic F108 (F108-OH), the intermediate α,ω-bismesylated Pluronic F108 derivative (F108-Ms), and the final α,ω-bisbenzaldehyded Pluronic F108 derivative (product) (F108-Bz) are illustrated in Figure S1 in the Supporting Information. The figure shows the complete end-functionalization of the F108-Ms and F108-Bz derivatives (end-group functionality of 2.0 for both).

#### Cross-Linker

As cross-linker for F108-Bz, we used our recently developed water-soluble tri(acylhydrazide) end-functional three-armed star oligo(ethylene glycol) with a glycerol core (AGE).^47^ The starting material was the corresponding trihydroxyl end-functional three-armed star oligoEG (triPEG) with a molar mass of 1000 g mol^–1^. The preparation required three steps, as in Deng’s original procedure.^44^ The first of these steps involved mesylation of the trihydroxy end-functional three-armed star oligomer at a 78% yield, subsequent attachment of methyl 4-hydroxybenzoate at an 88% yield, and finally, hydrazinolysis of the latter triester to obtain the corresponding trihydrazide (AGE) at a yield of 82%. This three-step procedure is illustrated in Figure S2.

### APCN Formation in Water

**Figure 2:**
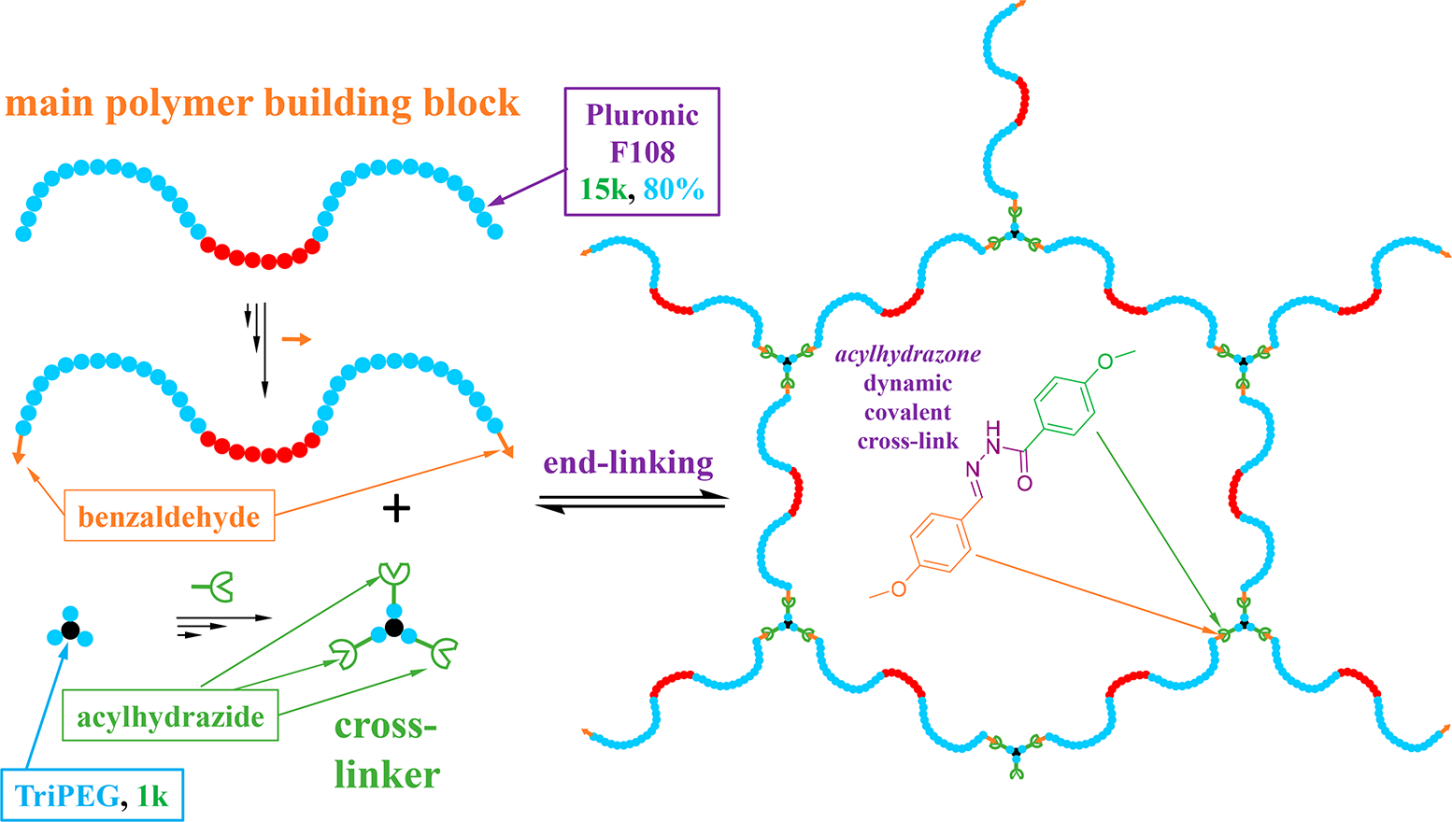
APCN gel formation procedure *directly in aqueous media* resulting from the mixing of the F108-Bz main building block and the AGE trifunctional cross-linker, each dissolved in a 100 mM acetate buffer at pH 4.5.

Figure 2 illustrates schematically the gel formation procedure resulting from the combination of the F108-Bz main polymer building block and the AGE TriPEG cross-linker *directly in water*. The two reagents were added at their stoichiometric ratio and at concentrations such that the final solids concentration (F108-Bz + AGE) was equal to 33% w/w. Due to the water-solubility of both F108-Bz and AGE (triPEG), the gel could be formed *directly in aqueous 100 mM acetate buffer of pH 4.5*; the acidity of the used buffer rendered gel formation possible without the need for the addition of catalyst, such as acetic acid. The gel formation times were on the order of seconds, too short to be determined by using the tube inversion technique or rheology.

### Temperature Dependence of the Viscoelastic Properties of the APCNs

**Figure 3:**
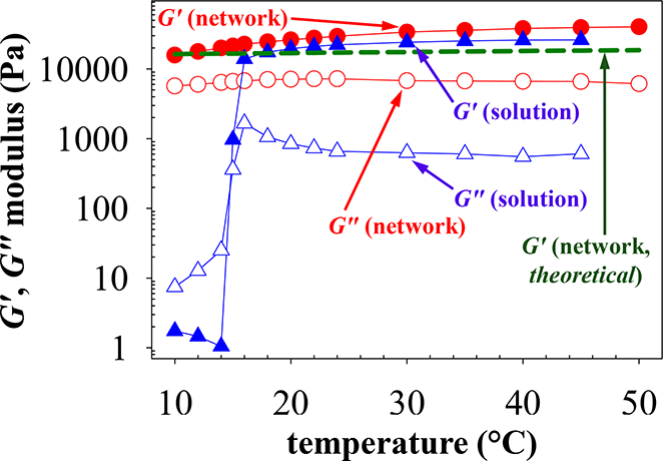
Dependence of the storage modulus (closed symbols), *G*′, and the loss modulus (open symbols), *G*″, on temperature for the as-prepared F108 APCN (red circles; chemically cross-linked network) and the F108-Bz amphiphilic polymer “solution” and resulting physical gel (blue triangles; physically cross-linked “solution”) both prepared in a 100 mM acetate buffer of pH 4.5 and at a final amphiphilic polymer concentration of 33% w/w, determined using oscillatory rheology at ω = 10 rad s^–1^ and γ = 1%.

Nonetheless, rheology was used to characterize the temperature-dependence of the viscoelastic properties of both the chemically cross-linked (as-prepared) gel (“network”, APCN) and the physical (thermally formed) gel (uncross-linked F108-Bz polymer “solution”), both containing an F108 concentration of 33% w/w in an aqueous buffer of pH 4.5. All results are overlaid and presented below in Figure 3 in which the storage modulus, *G*′, and the loss modulus, *G*″, are plotted against temperature for both systems.

The figure shows that the linear counterpart which can become only physically cross-linked (“solution”) presents a sol-to-gel transition at 14 °C, as manifested by the sharp increase in the value of the storage modulus, *G*′, at that temperature,^48,49^ becoming higher than the loss modulus, *G*″, despite the simultaneous increase of the latter modulus with temperature as well. Our determined critical gelation temperature of 14 °C is in excellent agreement with the literature value of 16 °C at the same polymer concentration.^48^ Gel formation in this nonchemically cross-linked system may be attributed to jamming^50^ arising from the close-packing of the block copolymer micelles^51^ (see section on APCN self-organization). In contrast, the APCN (“network”) was already a gel from the lowest explored temperature of 10 °C, as its *G*′ values were higher than the corresponding *G*″ values throughout the whole temperature range. In this case, both *G*′ and *G*″ slightly increased with the temperature. The main conclusion from this figure is that the dynamic covalent bonds in the APCN at the preparation pH held it together in the form of a chemical gel from the lowest applied temperature, whereas the uncross-linked linear counterpart became a physical gel only after its polymeric chains, and, their PG units, in particular, became hydrophobic enough to induce gel formation via jamming, which is the case just above 14 °C. According to literature, micellization at this polymer concentration already occurred from 6 °C. One might have expected to observe a distinct increase in the *G*′ value of the APCN at approximately 14 °C, as, at around that temperature, the physical cross-links being formed are added to the chemical ones. However, this was not the case, as the *G*′ value of the APCN gradually increased over the whole temperature range investigated. This might be due to the fact that the contribution from the physical cross-links to the modulus was smaller than that from the chemical ones. Indeed, at 16 °C, right after the physical gelation of the polymer solution, the *G*′ value of the physical gel was 11 kPa, as compared to a *G*′ value of 20 kPa for the APCN. It is noteworthy, however, that the difference in the *G*′ values became smaller at higher temperatures. In particular, the *G*′ values at 45 °C were 26 and 39 kPa for the physical and chemical networks, respectively. Thus, one may conclude that as temperature increases the APCN storage modulus *G*′ increasingly derives from jamming, with the contribution from the chemical cross-linking being smaller.

Figure 3 also plots the temperature-dependence of the theoretical storage modulus, *G*′, calculated from the (more realistic) phantom polymer network model according to eq 1,^3,52^ and assuming no micellization:
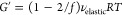
1where *f* is the functionality of the cross-linker, in our case 3 (trifunctional triPEG AGE cross-linker), ν_elastic_ is the theoretical molar concentration of the polymer elastic chains of 22.6 mM corresponding to the 33% w/w polymer mass concentration, *R* is the universal gas constant, and *T* is the absolute temperature.^3,52^ At 20 °C (*T* = 293 K), eq 1 above leads to the calculation of a theoretical *G*′ value of 18 kPa, which slightly increases to 20 kPa when the temperature is raised to 50 °C (*T* = 323 K). The theoretically calculated value of 18 kPa at 20 °C is very close to the experimentally determined *G*′ value for APCN at 16 °C of 20 kPa. In contrast, the theoretically calculated *G*′ value of 20 kPa at 50 °C is only half of the experimentally determined value at the same temperature of 40 kPa. The higher experimentally determined *G*′ value can be attributed to the fact that the APCN block copolymer components exist not as unimers, as assumed in eq 1 above, but rather as micelles with an aggregation number of about 85 (see the APCN self-organization section). If these micelles could be considered as star diblock copolymers, these stars would have an arm number of about 170 (since the constituting chains are ABA triblocks), essentially transforming eq 1 into the affine model.^3,52^ However, the right-hand side of this equation should be multiplied by a factor of 2/3 to take into account that each triPEG cross-link on average creates a double link between two adjacent micelles. This would give a *G*′ prediction of 40 kPa for the APCN at 50 °C, consequently matching perfectly the experimental *G*′ value at the same temperature.

### APCN Reversible Temperature Responsiveness

**Figure 4:**
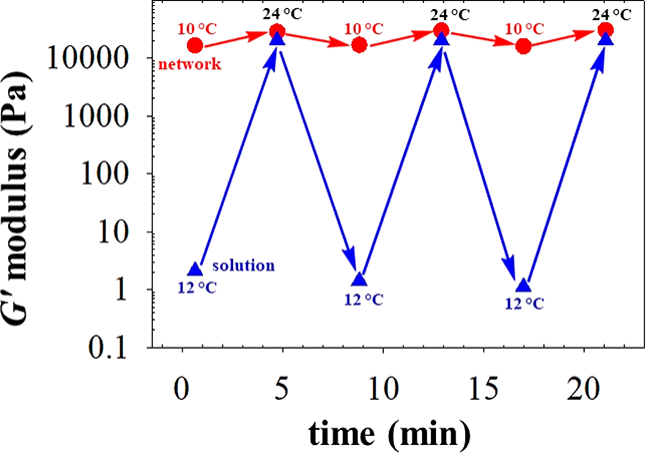
Response of the storage modulus upon temperature cycling between 10 (or 12) °C and 24 °C for the F108-based APCN and the corresponding un-cross-linked F108-Bz “solution” (giving a physical gel at 24 °C).

Figure 4 illustrates the results of temperature cycling experiments in rheology for both the chemical and physical gels at 33% w/w polymer concentrations. The experiments start from low temperatures, 10 or 12 °C, i.e., below 14 °C which is the sol-to-gel transition temperature at the given polymer concentration of 33% w/w, going up, within 5 min, to 24 °C, and then, again within 5 min, returning down to 10 or 12 °C. Consistently with Figure 3, Figure 4 shows that the particular temperature rise induces a dramatic increase in the storage modulus of the F108-Bz solution from about 2 to 20 200 Pa but only a modest increase in the storage modulus of the chemically cross-linked APCN from about 16 500 to 28 500 Pa. However, the new information that Figure 4 provides is that these increases can be thermally reversed totally within 5 min, as cooling from 24 °C down to 12 or 14 °C, with each of the two storage moduli regaining their initial low-temperature values. Furthermore, the figure shows that this cycling can be repeated for several cycles and is fully reversible. This reversibility indicates that the polymers and their cross-links (in the case of the APCN gel) do not undergo any (chemical or physical) damage during thermal cycling. Moreover, the changes are rather fast as they are completed within 5 min. Thus, the values of the storage moduli of the F108 gels highly depend on the nature of the cross-links, whether physical or chemical, and can be readily, swiftly, and reversibly modulated by temperature. The actual volume change was completed within tens of seconds (1–2 min), consistent with the temporal thermal-response of a model APCN system based on end-linked hydrophobic (thermoresponsive) and hydrophilic four-armed star homopolymers.^53^ Note that APCN swelling from the dried to the water-equilibrated state requires hours rather than minutes.^54^ Fast kinetics of APCN swelling/deswelling would be highly beneficial to solute diffusion when these networks are used as supports for bioatalysis.^55^

### Linear-Regime Thermorheological Properties and Dynamic Bond Lifetime

**Figure 5:**
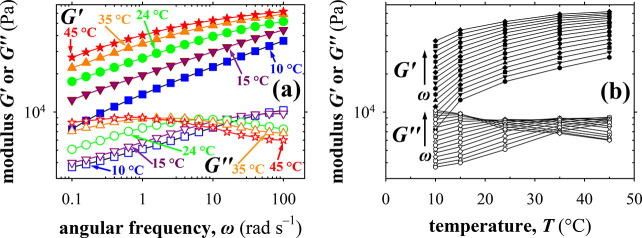
Dependence of the storage and loss moduli, *G*′ and *G*″, on (a) the angular frequency at different temperatures, 10, 15, 24, 35, and 45 °C, and (b) the temperature at different angular frequencies, ω, for the as-prepared F108-APCN formed at pH 4.5 and at a final amphiphilic polymer concentration of 33% w/w.

Next, we investigated the viscoelastic properties of the as-prepared APCNs formed at pH 4.5 and at a final F108-Bz amphiphilic polymer concentration equal to 33% w/w as a function of both the angular frequency, ω, and absolute temperature, *T*, using rheological frequency sweep experiments. The variations of ω and *T* were between 0.1 and 100 rad s^–1^ and between 10 and 45 °C, respectively. The viscoelastic properties explored were again the storage modulus, *G*′, and the loss modulus, *G*″. The dependence of *G*′ and *G*″ on ω and *T* is presented in Figure 5 whose part a focuses on the ω-dependence of the two moduli and part b concerns their *T*-dependence. Comparing the *G*′ and *G*″ values in the two parts of Figure 5, it may be observed that the *G*′ values are always above the corresponding *G*″ values without any crossing between the two quantities. Thus, bond lifetime cannot be determined from these results.

Figure 5a illustrates the dependence of the two moduli, *G*′ and *G*″, on ω at five different temperatures. The figure shows that *G*′ increases with ω as polymer chain relaxation increasingly lags behind the applied frequency. This trend is preserved with all five temperatures investigated. Furthermore, each G′ vs ω curve is shifted upward as temperature goes up, owing to the fact that the PG units become less hydrogen-bonded with water.^56^ The effect of temperature is more clearly depicted in Figure 5b where *G*′ is directly plotted against *T* (compare with Figure 3). In Figure 5b, *G*′ increases with *T*, and each *G*′ vs *T* curve is shifted upward as angular frequency goes up for the reasons already explained in the discussion of Figure 5a.

Figure 5a also illustrates the dependence of the loss modulus, *G*″, on ω at five different temperatures, whereas Figure 5b also exhibits the dependence of *G*″ on the temperature at different ω values covering three decades. Unlike the dependencies of *G*′ on ω and *T* in the same figure, which are always monotonic, the dependencies of *G*″ on ω and *T* in Figure 5 are more complex, as they are not always monotonic. In particular, at 10 and 15 °C, *G*″ increases almost linearly with ω in the double-logarithmic plot of Figure 5a, indicating a power-law dependence of *G*″ on ω and suggesting that, at higher temperature, the system increasingly dissipates energy due to its high mobility. The power-law exponent for these two temperatures is approximately 0.15, expectedly lower than the critical exponent of 0.56.^57^ An increase in temperature to 24 °C causes an initial increase in *G*″ with ω and, in particular, for ω values from 0.1 to 8 rad s^–1^, before it begins to steadily drop at higher ω values, denoting a reduction in the mobility of the system caused by diminishing hydrogen bonding between PPG and water.^56^ At 35 and 45 °C, the initial increase in *G*″ becomes less pronounced since the PPG aggregates are now less hydrogen-bonded with water, consequently affording lower energy dissipation. Finally, a complex behavior of *G*″ is depicted in Figure 5b, in which at the lowest ω values, *G*″ increases monotonically with *T*, at intermediate ω values *G*″ exhibits a maximum against *T*, and finally, at the highest ω values *G*″ decreases monotonically with *T*.

As mentioned in a previous paragraph, *G*′ was always higher than *G*″ in Figures 5, not allowing determination of the dynamic bond lifetime. Extending the experiments to lower frequencies produced noisy data, especially in *G*″. To obtain an estimation of bond lifetime, we resorted to our previous work on the tetraPEG gel homopolymer dynamic covalent system, based on the same cross-linking chemistry and for which we had performed frequency sweeps in a range of pH values.^43^ In that system, we obtained a clear moduli crossing at the very low pH of 1.5 and at a moderately low frequency. Subsequently, using the principle of superposition,^58^ we were able to estimate the frequencies of crossing for the systems at higher pH values, in which the moduli could not cross within a readily attainable frequency regime. The bond lifetime at pH 4.5 came out to be 9.3 h, whereas its graphical determination is given in Figure S3 in the Supporting Information.

### Tensile Mechanical Properties

**Figure 6:**
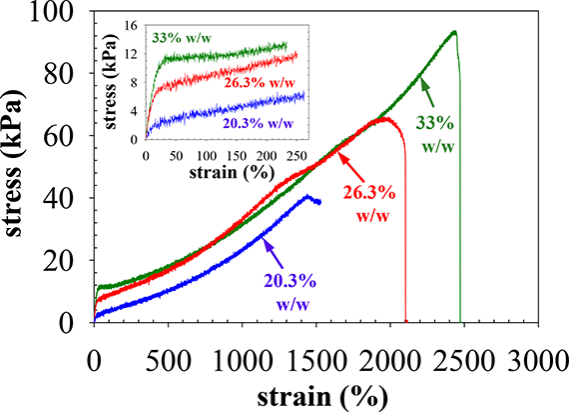
Representative tensile stress–strain curves for APCNs at different polymer concentrations.

**Figure 7:**
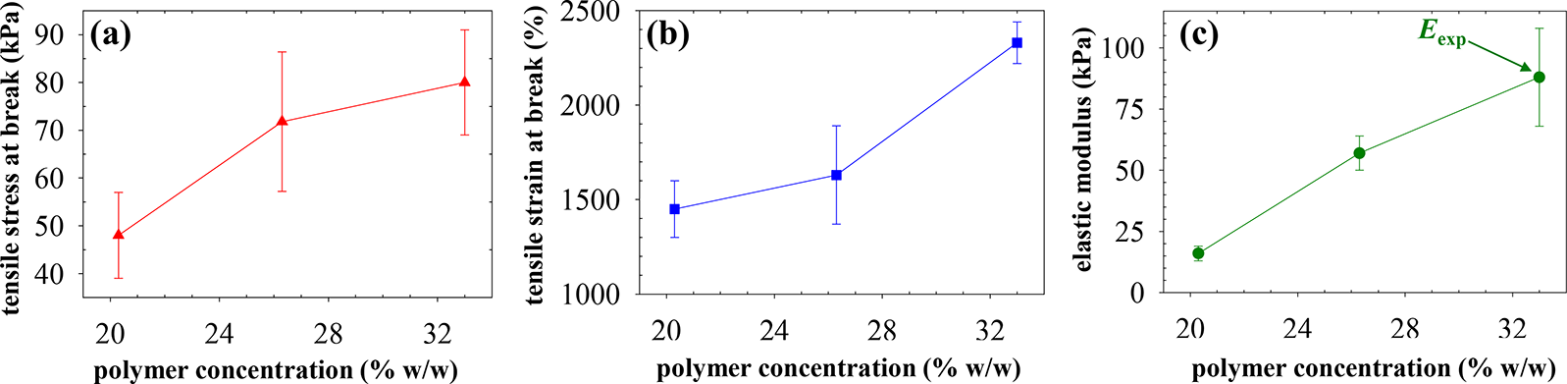
Amphiphilic polymer concentration dependence of (a) the tensile stress at break, σ_max_, (b) the tensile strain at break, ε_max_, and (c) the experimental, *E*_exp_, tensile Young’s (elastic) modulus of the as-prepared APCNs at pH 4.5. The measurements were conducted at room temperature.

In this section, we describe the characterization of the room temperature tensile mechanical properties of F108-based APCNs prepared at three different polymer concentrations, 20, 26 and 33% w/w. The mechanical properties determined were the tensile stress at break, σ_max_, the tensile strain at break, ε_max_, and the (experimental) tensile Young’s modulus, *E*_exp_. Representative stress–strain curves (for each polymer concentration, four to six measurements were performed on independently prepared samples) are illustrated in Figure 6, whereas the final results are plotted in Figure 7 where the error bars in the plots represent the standard deviations from the above-mentioned 4–6 repetitions.

Figure 6 shows that the stress–strain curves of the three APCNs prepared at different polymer concentrations present a slightly strain-hardening behavior until the points of failure, at rather low stress values, 35–90 kPa, but at remarkably high strains, 1500–2500%, depending on the polymer concentration. Focusing now on the tensile response at low strains, more clearly depicted in the inset to Figure 6, we may observe a rather sharp increase in stress with strain at very small strains, with the APCN at the highest concentration presenting a yield point, probably due to micellar unjamming, and also a characteristic of physically cross-linked networks and elastomers.^59^ The high initial slope in all three samples is followed by a shallower slope, corresponding to a modulus of ∼2 kPa for the two APCNs at the lowest polymer concentrations and a slope of almost 0 for the APCN at the highest polymer concentration suggestive of flow.

The curves end at the network failure point. Both the tensile stress and strain at break increase with the polymer concentration. Remarkably, the strain at break for the APCN prepared at the highest polymer concentration of 33% w/w is about 2400%, i.e., the sample broke only after it was stretched 23 times its initial length, even though it contained 67% w/w water. This is one of the most stretchable water-containing APCN samples reported in the literature.

All the results obtained after the averaging of all measurements at each concentration are presented in Figure 7, whose three parts illustrate (a) the tensile stress at break, (b) the tensile strain at break, and (c) the tensile Young’s modulus. All three quantities increase with the polymer concentration. The tensile stress at break for the most concentrated APCN reaches a good value of 80 kPa, whereas the tensile strain at break for the most concentrated sample goes up to an outstanding value of 2400%. Regarding *E*_exp_, its values increase with polymer concentration from 16 to 88 kPa. The enhancement of all three mechanical properties with APCN polymer concentration may be related to an increase in the micellar number density (24%) and micellar aggregation number (30%) with increasing block polymer concentration, as determined via extra SANS experiments (see Figure S4 and Table S1 in the Supporting Information).

### APCN Self-Healing and Self-Healing Efficiency in Tension

**Figure 8:**
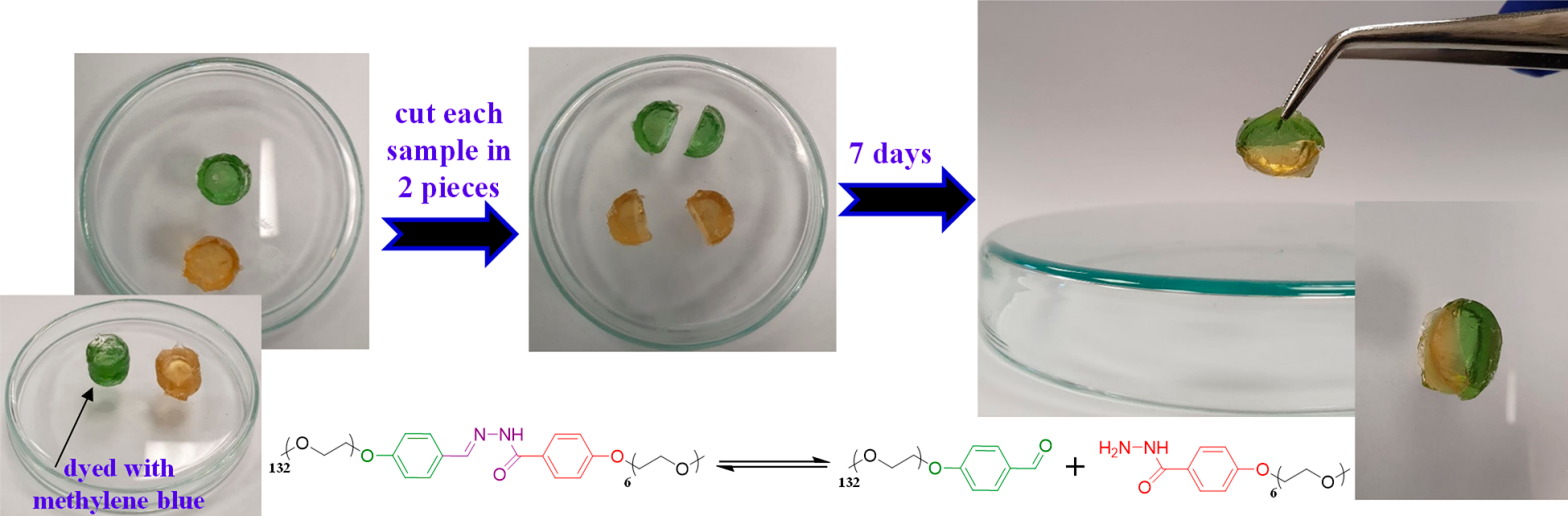
Self-healing of the APCNs prepared at a 33% w/w polymer concentration in aqueous buffer of pH 4.5. Two APCN samples were used, one of which was dyed with methylene blue (the other remained uncolored but possessing a yellowish own color). Each of the two samples was cut in half, and two different pieces were pressed against each other while keeping them at a low temperature (4–5 °C) without the addition of any self-healing catalyst.

The most important property of dynamic covalent gels is their ability to self-heal any damage they suffer. The self-healing ability of the presently developed APCNs is explored in this section, with the relevant experimental procedure illustrated in Figure 8. Two APCN samples were prepared at 33% w/w Pluronic F108 concentration in pH 4.5 aqueous buffer, one of which in the presence of a small amount of methylene blue dye that conferred to this sample a greenish color. Then, each sample was cut into two approximately equal pieces, and two different pieces, one from each original sample, were combined together by pressing them against each other for 7 days while in the fridge to keep the temperature low, at 4–5 °C (helps increase polymer solubility), but without the need for catalyst addition. The choice of a long mending time is justified from the long bond lifetime of almost 10 h estimated in the rheology section above. If a mending time equals an order of magnitude longer than bond lifetime, this gives almost 5 days, close to the 7 days elected as self-repair period. This procedure led to the joining of the two pieces together, arising from the breaking and re-formation of the hydrazone cross-links at the interface between the two different pieces; this exchange reaction is illustrated in the lower part of Figure 8. The self-healing efficiency was evaluated by subjecting the rejoined pieces to tensile testing. The results, given in Figure S5 in the Supporting Information, indicate a full recovery of the tensile strain at break and a near-quantitative, 93%, recovery of the tensile stress at break. Thus, the presently developed dynamically cross-linked materials are truly self-healable.

### APCN Self-Organization in D_2_O by SANS

**Figure 9:**
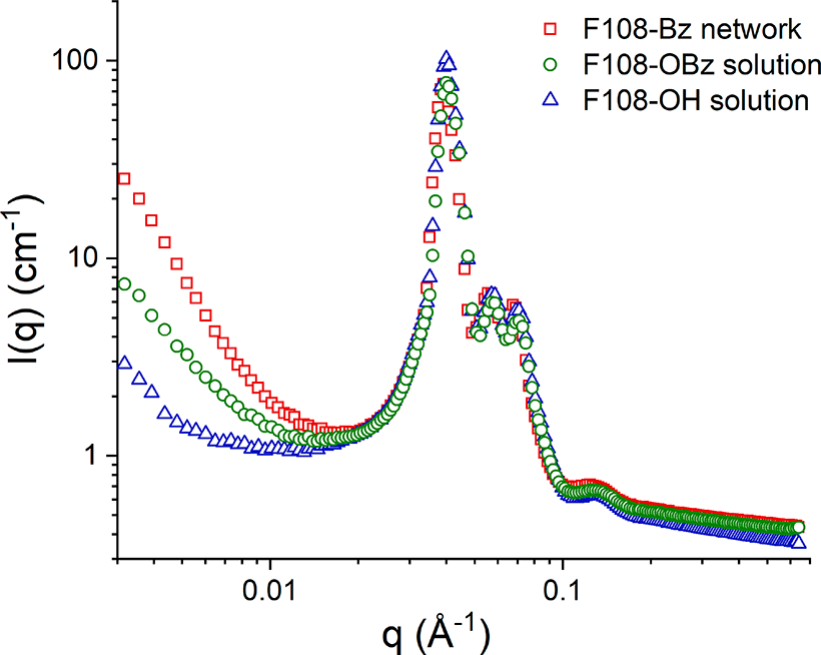
SANS intensity, *I*, as a function of the magnitude *q* of the scattering vector at room temperature (*T* = 24.7 °C) for the Pluronic F108-based APCN and its Pluronic F108-OH and Pluronic F108-Bz precursor solutions, all at 33% w/w polymer concentrations in D_2_O (pD = 4.5).

The most important property of amphiphilic block copolymer systems is their ability to self-assemble in solvents selective for one block. The self-assembly of the Pluronic F108-based APCN prepared at a 33% (w/w) polymer concentration in D_2_O (acetate buffer of pD = 4.5) was investigated using small-angle neutron scattering (SANS). The recorded SANS profile for this APCN is plotted in Figure 9, overlaid together with the SANS profiles of the original Pluronic F108-OH and the end-functionalized Pluronic F108-Bz in D_2_O buffer of pD = 4.5, also at a 33% w/w polymer concentration. The appearance of an intense main scattering peak in the SANS profiles of all three samples indicates that microphase separation has taken place. The average spacing between the scattering centers and the aggregation numbers of the F108 units calculated from the three SANS profiles are listed in Table 1.

**Table 1:** Results Obtained from the SANS Profiles of the APCN and the Two Solutions, All at Copolymer Concentrations of 33% w/w

no.	sample name	*q*_max_ (Å^–1^)	*d* (nm)	*N*_agg_
1	F108-Bz network	0.0391	22.73	86
2	F108-Bz solution	0.0407	21.82	76
3	F108-OH solution	0.0406	21.88	76

A first observation from the figure is that all of the scattering curves are almost indistinguishable from each other, except for the lower *q*-range, 0.003–0.010 Å^–1^. In this range, all three curves display an increase in the scattering intensity as *q* is lowered, with the highest increase exhibited by the APCN, followed by that from the F108-Bz, and the lowest increase presented by F108-OH. Nonetheless, all three scattering profiles in Figure 9 exhibit a main scattering peak at approximately the same value of the magnitude of the scattering vector, *q*, *q*_max_, and higher-order peaks, with ratios of their *q*-values relative to *q*_max_ equal to  and 2, with the last one being just visible as a shoulder on the previous scattering peak. The sharpness of the main peak and the appearance and sharpness of the higher-order peaks indicate a long-range ordering of the aggregates present.

The relative positions of the peaks indicate the presence of a body-centered cubic (BCC) structure, consistent with previous work that showed that Pluronics at higher concentration form cubic phases of a BCC structure,^60^ and this general observation was also confirmed for linear (un-cross-linked) Pluronic F108 in water for concentrations above 30 wt %.^61^ The un-cross-linked linear amphiphilic block polymer micelles can easily organize in solution with long-range order, but this high order could be lost upon their cross-linking into a network, as the constraints imposed by the cross-links could render the attainment of lowest free energy configurations with the minimization of the interfacial area very difficult.^37^ Thus, it is remarkable that the present APCN exhibits such a high degree of structural organization, whose high degree of ordering is unaffected by the chemical cross-linking. This may be attributed to the rather high molar mass of F108, approaching 15 kDa, whose relatively long chain, consisting of 314 monomer repeating units, may still allow for sufficient flexibility for optimal arrangement, even when cross-linked. Another possible reason for the observed microphase separation with long-range order is the dynamic nature of the cross-links, which exchange at the experimental acidic pH (pD) of 4.5. Through this exchange, the cross-links break and re-form within hours to days, with the partners on re-formation being potentially different from the ones before breaking, thereby allowing for further free energy minimization. This partner swinging is the same as the one mentioned in the previous section, which is responsible for self-healing (Figure 8).

The water-solubility of both APCN components enabled facile network formation in water, resulting in the obtained APCNs deriving directly from the F108 micellar state. Had network formation taken place in a nonselective organic solvent as in some of our previous work,^14^ followed by network transfer to an aqueous medium, APCN self-organization would not have been as orderly as that of the linear precursors in water. However, APCN self-organization would slowly (within a few days) be improving via dynamic cross-link exchange, eventually approaching that of its linear precursors or that of an APCN directly prepared in water.

For a BCC structure, one can calculate the aggregation number *N*_agg_ of the contained micellar aggregates from eq 2:^62^
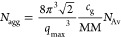
2where *c*_g_ is the weight concentration of the polymer, MM its molar mass, and *N*_Av_ the Avogadro constant. Table 1 lists the locations of the main scattering peaks, *q*_max_, the size *d* of BCC elementary cell calculated from *q*_max_ as 2π/*q*_max_, and the micellar aggregation numbers, *N*_agg_, calculated from eq 2 using the experimental polymer concentration. The *N*_agg_ value is then simply the number of block copolymer chains located within the unit cell cube, given by the product of the known molar copolymer concentration multiplied by the unit cell cube volume and taking into account the fact that the elementary BCC cell contains two micelles.^62^ It is important to point out that this calculation makes no assumptions about the degree of hydration of the PPG micellar core,^63^ and it is solely based on the distance among the scattering centers.

Finally, the higher increase of the SANS intensity at low *q* for the APCN, compared to those of its two precursor solutions, can be attributed to the network structure formed. Such a chemical network interconnects the scattering micellar aggregates over a longer distance, thereby leading to larger-scale inhomogeneities, and one sees this feature of the network structure by this increase at low *q*.

In addition, we also studied the effect of temperature on the different samples given in Figure 9. This was done by SANS measurements in the temperature range from 25 to 85 °C, and the resulting SANS curves for the different samples are given in Figures S7–S9 in the Supporting Information. All three samples exhibited the same temperature response with increasing temperature, where the intensity of the primary peak increased somewhat, the intensity at mid-*q* (0.005–0.03 Å^–1^) increased, most substantially for temperatures above 50 °C, and the higher-order correlation peaks largely vanished at the highest temperature of 85 °C, where, at the same time, the intensity at still higher *q* increased substantially. This may be interpreted such that the overall structure of the gels was slightly affected by temperature, especially with respect to the size of the hydrophobic domains, but with increasing temperature their degree of ordering became somewhat less pronounced, as evidenced by the disappearance of higher-order peaks and the increasing scattering intensity at lower *q*. However, it may be stated that the structure of the systems was remarkably stable with respect to changes in temperature, even at the highest temperature employed which is close to the cloud point of F108 in water.^46^

Table 1 shows that the values of all three above-mentioned quantities, *q*_max_, *d*, and *N*_agg_, are almost the same for the three samples studied. In particular, the *q*_max_ values are all around 0.04 Å^–1^, the size of the elementary cell is around 22 nm (which is expectedly much smaller, by a factor of 5, than the Pluronic F108 contour length of about 104 nm = 314 × 0.33 nm), and the *N*_agg_ values are around 80, thereby a bit higher than the values of 40–60 previously reported by dynamic light scattering for pure Pluronic F108 micelles.^64^ The fact that the cross-linked APCN gel self-assembled identically to its free (uncross-linked) counterparts can be attributed to the relatively long copolymer chains and their dynamic covalent end-linking, i.e., the same reasons as those to which gel structuring with long-range was attributed.

Furthermore, the effect of block copolymer concentration on the APCN self-organization properties was explored via extra SANS experiments presented in Figure S4 and Table S1 in the Supporting Information. As the F108 block copolymer concentration in the APCNs increased from 20.3 to 33.0% w/w, the intermicellar distance was slightly reduced from 24.4 to 22.7 nm, whereas the aggregation number increased from 66 to 86, manifesting a more densely packed micellar system at higher polymer concentration, capable of better mechanical performance (also see Figures 6 and 7).

### Use as Matrix for Gel Polymer Electrolytes

Finally, the Pluronic F108-based APCN system was evaluated as a matrix for the fabrication of gel polymer electrolytes (GPEs) to be used as ion-conducting and -separating membranes in lithium ion batteries (LIB). The GPEs investigated here comprise the dynamically cross-linked Pluronic F108 conetwork swollen in an appropriate ionic liquid but without water or organic solvent. The employed ionic liquid was a commercial mixture from Solvionic consisting of two organic salts, lithium bis(trifluoromethanesulfonyl)imide (LiTFSI) and 1-ethyl-3-methylimidazolium bis(trifluoromethanesulfonyl)imide (EMIM-TFSI), combined at a 1:9 molar ratio. The F108-based GPEs were prepared by combining acetonitrile solutions of the dibenzaldehyde end-functionalized Pluronic F108 ABA triblock copolymer (F108-Bz) with the acylhydrazide end-functionalized Gly-TriPEG(1000) cross-linker (TriPEG-ac or AGE), in which the F108-Bz solution also carried the ionic liquid, whereas the TriPEG-ac solution contained a cross-linking catalyst, acetic acid. A single GPE formulation was fabricated to match the solvent content of the APCN hydrogel previously investigated in this manuscript, which, after acetonitrile and acetic acid evaporation at 40 °C under vacuum, contained 33% w/w polymer and 67% w/w ionic liquid mixture. Furthermore, each GPE membrane had a thickness in the range of 150–200 μm.

Before proceeding to electrochemical characterization, we characterized the developed GPE in terms of its tensile mechanical properties. This was necessary as one of the planned experiments involved the electrochemical characterization of the GPE in tension. Figure S6 presents two duplicate stress–strain curves of the GPE, which were very close to each other; the same figure also displays the calculated average tensile mechanical properties (Young’s modulus, stress, and strain at break) together with the corresponding standard deviations. While the tensile strain at break in the aqueous APCN system was about 2400% (Figure 7b), the ionic-liquid-containing system could only be stretched about half that value before fracturing. Thus, the electrochemical characterization of the stretched GPE was performed at a strain varying between 100 and 1000%. We then proceeded to the electrochemical characterization of the GPE in its native, stretched, and recycled states.

**Figure 10:**
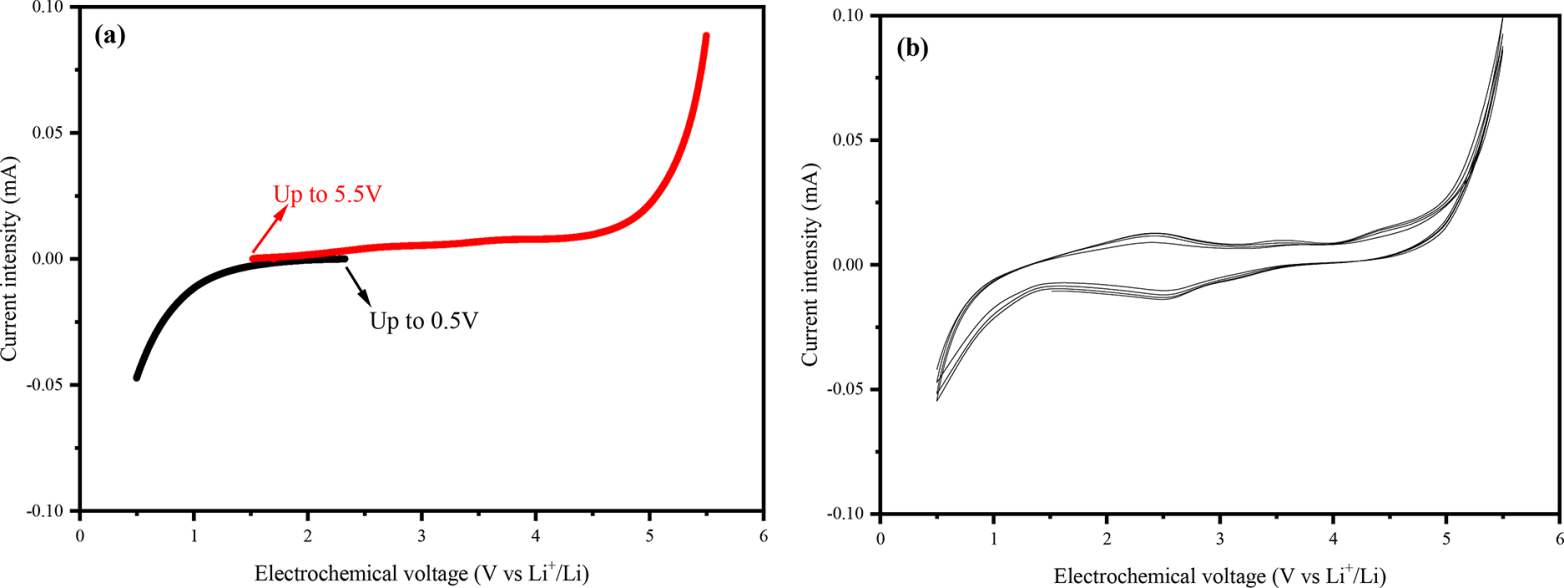
Electrochemical stability of the F108-based APCN GPE between 0.5 and 5.5 V vs Li^+^/Li at room temperature: (a) linear sweep voltammetry of two CR2025 cells from the open circuit voltage to 0.5 V (in black) and to 5.5 V vs Li^+^/Li (in red); (b) a four-scan cyclic voltammetry between 0.5 and 5.5 V vs Li^+^/Li.

To be compatible with LIB technologies, our GPE system should be stable at low and high voltages. Consequently, we studied the electrochemical stability between 0.5 and 5.5 V vs Li^+^/Li, and the results are plotted in Figure 10. For this, the F108 APCN GPE was sandwiched between a stainless-steel disk (onto which the membrane was cast) and a lithium chip of 15 mm diameter. Another stainless-steel disk was added as a spacer, and the whole assembly was sealed in a CR2025 coin cell. Linear sweep voltammetry (Figure 10a) suggests no important redox reactions between 1 and 5 V vs Li^+^/Li. However, cyclic voltammetry (Figure 10b) shows a small, highly reversible peak around 2.5 V vs Li^+^/Li. Moreover, the oxidation peak observed above 5.0 V can be attributed to the ionic liquid, indicating that this GPE is definitely suitable for conventional Li-ion batteries (3.0–4.2 V vs Li^+^/Li) and possibly suitable for high potential lithium–nickel–manganese–cobalt oxides (NMC) Li-ion batteries (up to 4.8 V vs Li^+^/Li).

Potentiostatic electrochemical impedance spectroscopy (PEIS) was used to characterize the ionic conductivity of the GPE systems and was performed following a program of decreasing temperature from 80 down to 20 °C, in 10 °C steps, and with a 2 h rest at each step. To ensure the reproducibility of the results, six extra GPE samples were prepared on stainless steel disks and analyzed by PEIS, with the measurement on each sample being repeated three times. All recorded impedance spectra corresponded to a line without semicircle, which is typical of systems where only ion diffusion leads to a resistance, and no semicircle at high frequencies which would have been associated with a supplementary interface in the system, as a solid component, a nonhomogeneous mixture or a crystalline phase. Typical impedance spectra obtained for these GPEs are shown in Figure S10 illustrating the spectra corresponding to the stretched (strain of 1000%) GPEs.

**Figure 11:**
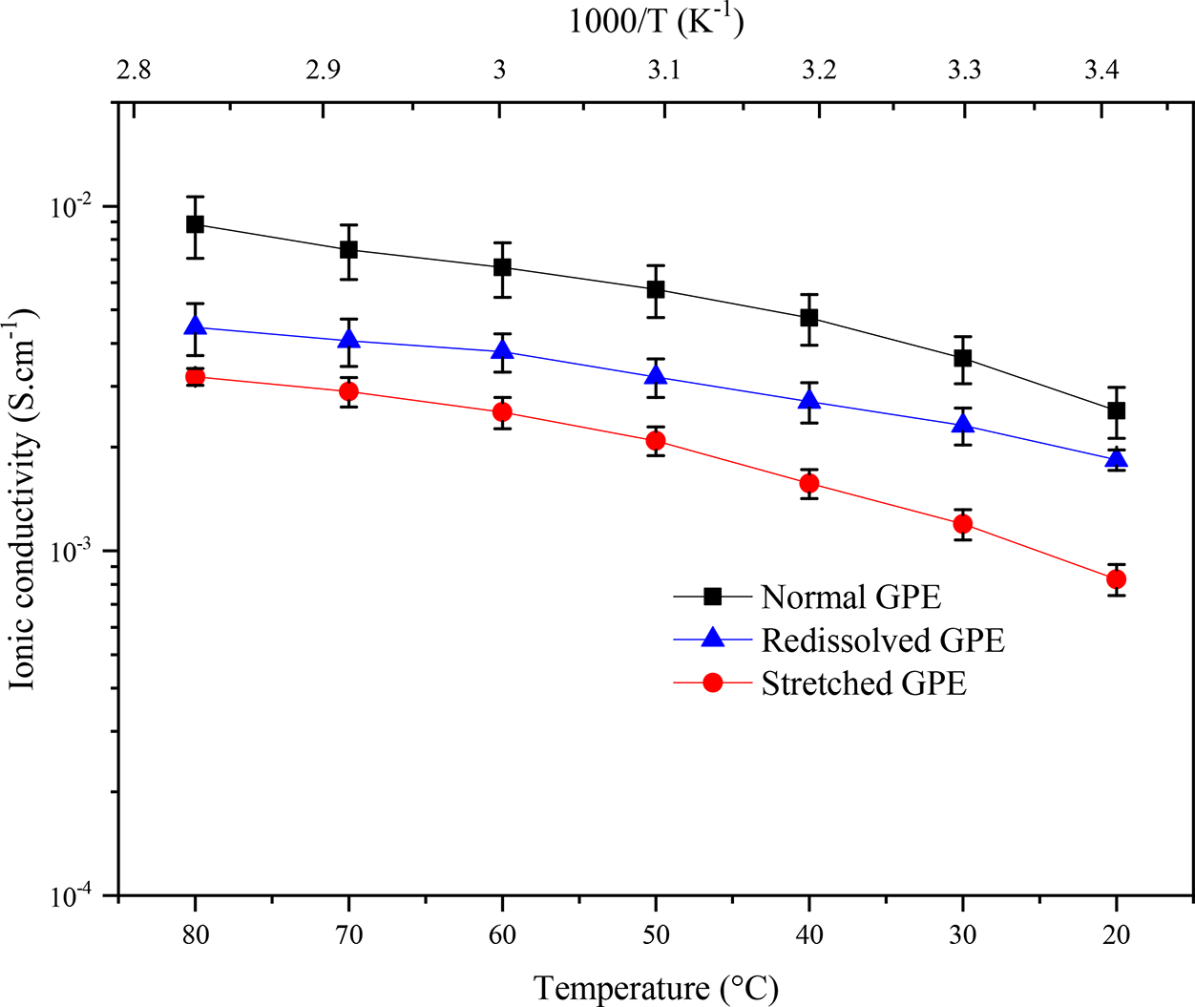
Temperature dependence of the F108-based APCN GPEs ion conductivity (“setup 1”) for normal gel (black), after dissolution and regelation (blue), and after a 1000% stretching (red). Error bars are based on three sets of data.

Figure 11 displays the temperature-dependence of the ion conductivity for the three different GPE systems (three samples were measured for each system to ensure reproducibility of the results), while the data obtained on the six extra investigated normal GPE samples are displayed in Figure S11. The ionic conductivity, σ, was calculated according to eq 3:
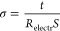
3where *t* is the thickness (in cm) of the sample at the end of the analysis, *R*_electr_ is the electrical resistance (in Ω) of the sample measured using PEIS, and *S* is the surface area (in cm^2^) of the sample.

The conductivity values ranged from about 3 mS cm^–1^ at room temperature (20 °C) to 10 mS cm^–1^ at 80 °C for the genuine GPE, similar to the ones exhibited by polar homopolymer-based (tetraPEG gel) GPEs.^65,66^ Interestingly, the room temperature ion conductivity of the GPE was rather close to the corresponding value for the pure 1:9 LiTFSI–EMIM-TFSI IL (6 mS cm^–1^). This result is in sharp contrast to the much lower room temperature ion conductivity of 0.5 mS cm^–1^ measured for our previously studied APCN system based on the combination of four-armed poly(vinylidene fluoride) star homopolymer (tetraPVDF) and four-armed poly(ethylene glycol) (tetraPEG) star homopolymer,^14^ where the latter star homopolymer component is PEG, which is the main component of Pluronic F108. That is, the two APCN systems are chemically very similar, as they both contain PEG segments. The low room temperature ion conductivity of our previously studied tetraPVDF-tetraPEG system could be related to the PVDF hard component, which reduced overall chain mobility. In contrast, the presently developed F108 APCN system possesses very mobile chains, as manifested by its very high extensibility of ∼1000% in the ionic liquid environment, indicating extensive chain relaxation which may facilitate ion diffusivity.

In a further step, the GPE samples were stretched before PEIS measurements. Two different procedures (“setups”) were implemented to investigate the ion conductivity of stretched samples. In both types of experiments, the stretching was performed on GPE membranes initially prepared in a Teflon mold to allow for facile removal for subsequent membrane handling. In the initial experiments, the GPE membranes were stretched to 1000%, their thickness was measured, and the stretched membranes were further sandwiched between two stainless steel disks and then placed in a coin-cell case for ionic conductivity measurements in temperatures ranging from 80 down to 20 °C (“setup 1”). The advantage of this procedure is that it is the same as the one used for the genuine, nonstretched sample, and it easily allows ionic conductivity measurements at varying temperatures by placing the coin-cell in a temperature-controlled chamber. The main disadvantage of this method is that further control of the sample thickness is no longer possible after the membrane is placed in the coin-cell. However, changes in thickness may occur due to possible relaxation of the stretched membrane, which would affect the calculation of ionic conductivity. The results obtained by this method are also plotted in Figure 11, and indicate a systematic reduction in the calculated ionic conductivity of the stretched GPE membranes compared to the genuine sample.

**Figure 12:**
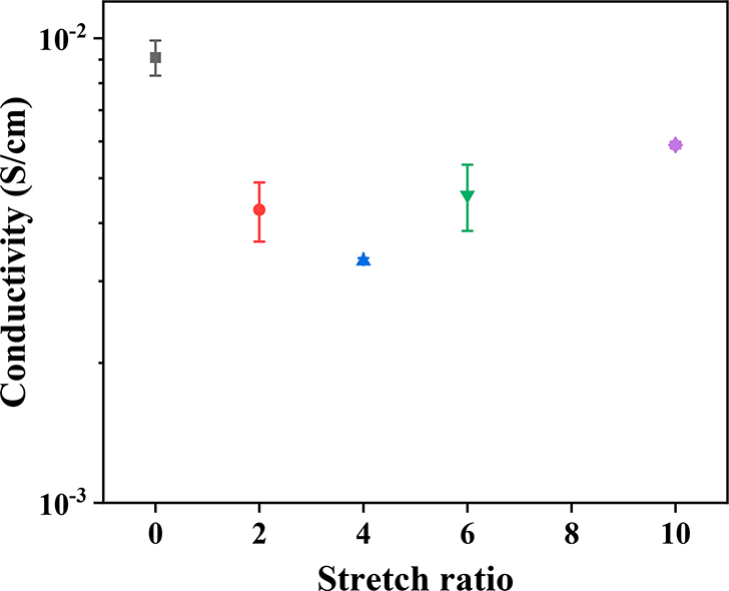
Evolution of ionic conductivity at room temperature of GPE samples stretched to different degrees, as measured using “setup 2”.

Because of the uncertainties related to possible membrane thickness variations, another experimental procedure was devised and implemented for the ionic conductivity measurements. Here, the GPE membranes detached from the Teflon mold were placed on a copper film for stretching (“setup 2”) (see Figure S12). The ionic conductivity and film thickness measurement equipment in “setup 2” was fitted together in a glovebox to also allow precise determination of film thickness during the PEIS measurements, circumventing the main drawback of the previous method (“setup 1”). Nevertheless, only measurements at room temperature were possible within “setup 2”, since the utilized equipment was located in a glovebox without temperature control. Different degrees of stretching were examined. Stretching degrees were varied from 100% (2-fold stretched sample) to 1000% (10-fold stretched sample), and the stretching process was repeated twice to check reproducibility. In agreement with the results initially obtained with the first experimental setup, “setup 1”, all stretched GPE systems characterized using “setup 2” also displayed lower ionic conductivity at room temperature compared to the nonstretched counterpart (see Figure 12). However, the decrease in ionic conductivity values was less pronounced with this second setup compared to the initial one. Furthermore, the ionic conductivity measured using “setup 2” was minimized at 4-fold stretching, presenting a gradual increase for higher stretching degrees, but without reaching the ionic conductivity of the genuine, nonstretched sample.

Our results with an initially decreasing ion conductivity with increasing strain may simply be attributed to the increasing barrier to through-plane (the direction in which ion conductivity was measured and was perpendicular to the direction of stretching) Li-ion diffusion (considering that conductivity predominantly arises from the LiTFSI–EMIM-TFSI ionic liquid swelling the F108-based polymer network and less from ion hopping on the polymer chains) imposed by the stiffened polymer chains which are oriented along the stretching direction. The above-invoked diffusion barrier is analogous to the submicrometer “grain” structure identified in other block copolymer (albeit non-cross-linked) electrolyte systems, in which intragrain ion diffusion is fast, with intergrain ion diffusion being slow and rate-limiting.^67,68^ Moreover, in such a grain-composed system, upon stretching, the constituting grains should be deformed from an initially spheroidal shape to a prolate-ellipsoidal one with the major axes of the ellipsoids aligned along the direction of stretching.^69^ With this geometry, one may expect that ion diffusion and ion conductivity would be enhanced in the direction of stretching (“in-plane” ion conductivity)^70^ because the path of intragrain ion transport is lengthened,^69^ whereas ion diffusion and ion conductivity would be reduced in the perpendicular direction (“through-plane” ion conductivity)^67^ because the path of intragrain ion transport is shortened and slow intergrain transport becomes dominant.^69^ Finally, the increase in (through-plane) ionic conductivity at strains above 4 may indicate that these higher degrees of stretching create Li-ion diffusion pathways. These pathways may result from cracks formed in these highly stretched gels, thereby disrupting intergrain boundaries.

Lastly, we observed a decrease of about 50% of the initial ion conductivity when the GPE was dissolved and re-formed again, to reach 1.4 and 4.4 mS cm^–1^ at 20 and 80 °C, respectively (also plotted in Figure 11). This could be attributed to the lower weight percentage of polymer in the solution needed to dissolve the gel before casting it again, which can lead to a not perfectly homogeneous gel or a less cross-linked network, although the adventitious introduction of impurities into the system cannot be excluded. Nevertheless, this also shows that this system can be easily stretched or redissolved and still leads to very acceptable ionic conductivities of >1 mS cm^–1^ at room temperature.

## Conclusions

Herein, we have presented the development of a new amphiphilic polymer conetwork (APCN) system with several important properties and great potential for use in a timely energy-related application. These properties included self-organization into a BCC structure with long-range order, great stretchability up to 2500%, and self-healing ability with near-quantitative recovery of the tensile mechanical performance. When loaded with an ionic liquid mixture, this APCN system constitutes a gel polymer electrolyte (GPE), appropriate for use as the separating membrane in lithium ion batteries. This GPE is electrochemically stable and maintains a high ion conductivity, reasonably close to that of the carried ionic liquid mixture. Upon elongation, the through-plane ion conductivity of this GPE is initially reduced and then increases, due to ion diffusion through shorter intragrain paths and due to grain boundary disruption, respectively.

## Supporting Information Available

Experimental section with materials and synthesis (end-functionalizations, APCN and GPE preparations) and characterization methods (rheology, tube inversion, ^1^H NMR spectroscopy, self-healing, mechanical testing, SANS, ion conductivity, and electrochemical stability measurements), supporting figures including ^1^H NMR spectra, synthetic schemes, mechanical testing of GPEs and self-healed APCNs, temperature-dependence of SANS profiles of the APCN and its linear precursors, and polymer concentration-dependence of SANS profiles of APCNs (PDF)
