# Supplementary material for: Dynamic Covalent Amphiphilic Polymer Conetworks Based on End-Linked Pluronic F108: Preparation, Characterization, and Evaluation as Matrices for Gel Polymer Electrolytes
Source: ACS Appl Mater Interfaces. 2024 Apr 26;16(18):23813–25. doi: 10.1021/acsami.3c19189 (PMC11082838; doi:10.1021/acsami.3c19189)
Supplement: Supplementary file 1 — am3c19189_si_001.pdf [file am3c19189_si_001.pdf]

Supporting Information

Dynamic Covalent Amphiphilic Polymer Conetworks Based on End-linked Pluronic F108: Preparation, Characterization and Evaluation as Matrices for Gel Polymer Electrolytes

by Demetris E. Apostolides,[1] George Michael,[1] Costas S. Patrickios,[1,]* Benoit Notredame,[2] Yinghui Zhang,[2] Jean-François Gohy,[2]
Sylvain Prévost,[3] Michael Gradzielski,[4] Florian A. Jung[5] and Christine M. Papadakis[5]

Submitted for publication in *ACS Applied Materials & Interfaces*

December 2023

Revised: April 2024

Accepted: April 2024

[1] Department of Chemistry, University of Cyprus, P. O. Box 20537, 1678 Nicosia, Cyprus

[2] Institute for Condensed Matter and Nanosciences (IMCN), Bio- and Soft Matter (BSMA), Université catholique de Louvain (UCL), Place Pasteur 1, 1348 Louvain-la-Neuve, Belgium

[3] Institut Max von Laue - Paul Langevin (ILL), 71, avenue des Martyrs - CS 20156, 38042 Grenoble, cedex 9, France

[4] Stranski-Laboratorium für Physikalische und Theoretische Chemie, Institut für Chemie, Straße des 17. Juni 124, Technische Universität Berlin, D-10623 Berlin, Germany

[5] TUM School of Natural Sciences, Physics Department, Soft Matter Physics Group, Technical University of Munich, James-Franck-Str. 1, 85748 Garching, Germany

* Correspondence to: Costas S. Patrickios, **e-mail:** costasp@ucy.ac.cy

Contents

page

Title, Authors' Names and Addresses **1**

Contents **2**

Experimental Section .... **3 – 8**

Materials and Methods .... **3 – 8**

Materials 3

Synthetic Methods .... **3 – 5**

Synthesis of the di-mesylate-terminated-PEG-*b*-PPG-*b*-PEG (F108-Ms) … 3 – 4

Synthesis of the di-benzaldehyde-terminated-PEG-*b*-PPG-*b*-PEG (F108-Bz) .. 4

Preparation of the F108-based dynamic covalent APCN (F108-APCNs) …... 4

Preparation of the F108-based gel polymer electrolyte (F108-GPE) …….. 4 – 5

Characterization Methods .... **5 – 8**

Rheology .... 5 – 6

Tube inversion 7

Proton nuclear magnetic resonance (^{1}H NMR) spectroscopy…………… 7

Self-healing experiments 7

Tension measurements 7

Small-angle neutron scattering 8

Ionic conductivity measurements 8

Electrochemical stability measurements 8

Supporting Figures .... **9 – 15**

References **16**

Experimental Section

Materials and methods

Materials. Pluronic F108 [*α*,*ω*-dihydroxy-poly(ethylene glycol)-*b*-poly(propylene glycol)-*b*-poly(ethylene glycol) (HO-PEG-*b*-PPG-*b*-PEG-OH) ABA triblock copolymer with 80 wt.% PEG and average molar mass of 14600 g mol^{-1} (F108-OH), diethyl ether (purity ≥ 99%), 4-hydroxybenzaldehyde (98%), methanesulfonyl chloride (≥ 99.7%), calcium hydride (CaH_2, 90–95%), sodium carbonate (≥ 99%), anhydrous 1,4-dioxane (99.8%), anhydrous magnesium sulfate ($MgSO_4$, ≥ 99.5%), potassium carbonate (K_2CO_3, ≥ 99%), sodium acetate (≥ 99%), glacial acetic acid, sodium hydroxide (≥ 98%), dichloromethane (DCM, 99%), *N,N*-dimethylformamide (DMF, 99.8%), triethylamine (TEA, ≥ 99%), and deuterated chloroform ($CDCl_3$, 99.8%) were purchased from Sigma-Aldrich-Fluka-Merck, Germany. An ionic liquid (IL) electrolyte, comprising a mixture of two organic salts, lithium bis(trifluoromethanesulfonyl)-imide (LiTFSI) and 1-ethyl-3-methylimidazolium bis(trifluoromethanesulfonyl)-imide (EMIM-TFSI), combined at a 1 : 9 LiTFSI : EMIM-TFSI molar ratio, was purchased from Solvionic in Toulouse, France. Tri-benzaacylhydrazide-terminated tri-arm star poly(ethylene glycol) of M_n = 1402 g mol^{-1} (AGE) was prepared according to our previously published procedure [1]. An aqueous acetate buffer solution (100 mM) of pH 4.5 was prepared from glacial acetic acid and sodium acetate.

Synthetic Methods. *Synthesis of the di-mesylate-terminated-PEG-b-PPG-b-PEG (F108-Ms)*. First, F108-OH (5 g, 0.342 mmol) was dissolved in a minimal amount of 1,4-dioxane and freeze-dried under vacuum. Afterward, the dried F108-OH was dissolved in DCM (30 mL) freshly distilled from CaH_2, and then, TEA (0.6 mL, 7.5 mmol), again freshly distilled from CaH_2, was added into the polymer DCM solution. After that, the mixture was cooled down to 0 °C, and methanesulfonyl chloride (0.6 mL, 0.86 g, 7.5 mmol) was added dropwise under stirring. Then, the mixture was left at room temperature for three days under continuous stirring. Subsequently, the mixture was diluted with DCM, washed eight times with saturated aqueous solution of sodium carbonate, and twice with deionized water. Next, the organic phase was dried over anhydrous $MgSO_4$, filtered, condensed under reduced pressure, and precipitated twice in cold diethyl ether. In the end, the final precipitate was dried under vacuum at 50 °C. The F108-Ms was isolated as a yellowish powder at 77% yield (3.85 g, 0.261 mmol).

Synthesis of the di-benzaldehyde-terminated-PEG-b-PPG-b-PEG (F108-Bz). F108-Ms (2.5 g, 0.17 mmol), 4-hydroxybenzaldehyde (0.31 g, 2.5 mmol), K_2CO_3 (0.35 g, 2.5 mmol), and DMF (30 mL) freshly distilled from CaH_2 were transferred into a round-bottomed flask. Afterward, the reaction mixture was refluxed under stirring for 24 h. After that, the mixture was diluted with DCM, washed eight times with a 0.75 M aqueous solution of sodium hydroxide, and twice with deionized water. Next, the organic phase was dried over anhydrous $MgSO_4$, filtered, condensed under reduced pressure, and precipitated twice in cold diethyl ether. In the end, the final precipitate was dried under vacuum at 50 °C. The F108-Bz was isolated as a brown powder at 73% yield (1.82 g, 0.123 mmol).

Preparation of the F108 dynamic covalent APCN (F108-APCN). The F108-APCNs were prepared by mixing acetate (0.1 M) aqueous buffered solutions at pH 4.5 of F108-Bz and the low molar mass AGE cross-linker at their stoichiometric ratio. The F108-Bz concentration in the mixture was kept constant at 33% w/w. The general procedure followed for the preparation of the F108 APCNs used in rheology, self-healing, and tension experiments is given below. First, F108-Bz (0.263 g, 0.17 mmol) and 470 μL of the aqueous acetate buffer of pH 4.5 were transferred into a glass vial. After that, the temperature of the glass vial and its contents was adjusted to 4-5 °C (for 2 days) to facilitate the dissolution of the copolymer. Subsequently, the cold F108-Bz solution was mixed with 50 μL of an aqueous acetate buffer solution of AGE (16.6 mg, 11.8 μmol). Then, the mixture was left for one week in the refrigerator at 4-5 °C and allowed to form a gel.

Preparation of the F108-based gel polymer electrolyte (F108-GPE). The F108-based GPEs (F108-GPE) were prepared by combining acetonitrile solutions of F108-Bz and AGE at their stoichiometric ratio. The F108-Bz solution also carried the LiTFSI – EMIM-TFSI ionic liquid (IL), whereas the AGE solution carried 5% v/v glacial acetic acid. A single F108-based GPE formulation was fabricated, which, after acetonitrile and acetic acid evaporation, contained 33% w/w polymers and 67% w/w IL. Details of the preparation follow.

Two stock solutions were prepared. The first solution (Sol1) was prepared by adding 0.75 g of F108-Bz (M_n = 14 808 g mol^{-1}) into 1442 μL acetonitrile, followed by stirring for 2 h to completely dissolve the polymer. The second stock solution (Sol2) was prepared by adding 0.05 g of tris(acylhydrazide)-PEG (M_n = 1402 g mol^{-1}) into 96 μL acetonitrile, followed by stirring for 2 h to completely dissolve the AGE cross-linker. The F108-GPE was prepared by mixing 360 μL of

Sol1 with 676 µL of the IL on the one side, and 22.4 µL of Sol2 with 55.6 µL of acetic acid on the other side. Each solution was vortex-mixed for 5 min, and was then added together to be finally vortex-mixed again for 2 min. The obtained solution was then cast on 8 stainless steel disks (15.5 mm diameter, 0.5 mm thickness) and was allowed for at least 24 h to proceed to gel formation. The obtained gel was then dried under high vacuum (0.5 mbar) at 40 °C for 24 h so that acetonitrile and acetic acid could evaporate. The final gel thickness was around 200 µm. One sample was cast on a PTFE cup to be stretched 10 times its size (from 0.5 to 5 cm in length). All this procedure was performed in an argon glove box with less than 0.1 ppm of H_2O and O_2.

For the recycled F108-GPE, after analysis, the sample (0.11 g) was mixed with 700 µL of acetonitrile over 3 h. Then 35 µL of acetic acid (5% v/v) was added and the solution stirred for 10 min before casting on a stainless steel disk (15.5 mm diameter, 0.5 mm thickness). The mixture was left at rest for 24 h so that it could form a gel. The resulting gel was then dried under high vacuum (0.5 mbar) at 40 °C for 24 h so that acetonitrile and acetic acid could evaporate.

Characterization Methods. *Rheology*. Rheological measurements were performed on the F108-based APCN and the aqueous F108-Bz solution, both buffered at pH 4.5, using a Discovery HR2 rheometer from Thermal Analysis Instruments (TA), operating in parallel plate geometry. The top plate had a 20 mm diameter, whereas the bottom plate was a Peltier element, thermostatically controlling the system. Moreover, a solvent trap was used to prevent any evaporation of the solvent. Three types of temperature-ramp experiments were performed, all at a 1% strain. The first two types of temperature-ramp experiments were in the oscillation time mode adopting a constant angular frequency of 10 rad s^{-1}, whereas the last one was in the oscillation frequency mode with the angular frequency increasing from 0.1 to 100 rad s^{-1}.

The first type of temperature-ramp rheological experiments in the oscillation time mode covered the temperature range from 10 to, typically, 50 °C in 2 or 5 °C temperature increments. In the following, we provide the details for the characterization of the F108-Bz solution using this first type of rheological experiment. In the beginning, the 33% w/w F108-Bz aqueous solution was cooled down to ~ 5 °C in the refrigerator, and was, subsequently, transferred onto the top plate of the rheometer. Afterward, the upper plate was lowered down to a certain gap, the solvent trap was adjusted, and the temperature of the Peltier plate was set at 10 °C. After 3 min, the operation of the rheometer was initiated in the oscillation time mode for 1.5 min. Then, the temperature was

increased to 12 °C and the F108-Bz solution was left for 3 min to equilibrate, before the next temperature measurement. The previous experimental steps were repeated at higher temperatures, that increased in 2 to 5 °C intervals, up to 45 °C. The whole temperature-ramp oscillation time mode rheology experiment was also performed on the F108-APCN, within the temperature range from 10 to 50 °C, again in 2 to 5 °C increments.

In the second type of temperature-ramp experiments in the oscillation time mode, the rheometer was operated at only two temperature values, 12 (or 10) and 24 °C, with the temperature being increased from 12 (or 10) to 24 °C, and then being lowered down to 12 (or 10) °C, with the cycle being repeated three times. Each sample was allowed to equilibrate for 3 min at each temperature. The 33% w/w F108-Bz aqueous solution was cycled between 12 and 24 °C, whereas the corresponding F108-APCN was cycled between 10 and 24 °C. The slightly lower "cold" temperature for the F108-APCN was chosen on the basis of its slightly higher anticipated hydrophobicity in comparison to that of its "solution" counterpart.

The last type of temperature-ramp experiments were in the oscillation frequency mode, and concerned only the F108-APCN. This experiment employed an initial frequency of 0.1 rad s^{-1}, which gradually increased to 100 rad s^{-1}, and this whole frequency scan was successively performed at 10, 15, 24, 35 and 45 °C. In the following, we provide some details on the characterization of the F108-APCN using this third type of rheological experiment. In the beginning, the F108-APCN was transferred onto the top plate of the rheometer. Afterward, the upper plate was lowered to a certain gap, the solvent trap was adjusted, and the temperature of the Peltier plate was set at 10 °C. After 3 min, the operation of the rheometer was initiated in the oscillation frequency mode, covering the angular frequency range from 0.1 to 100 rad s^{-1}. Next, the temperature was set to 15 °C, the F108-APCN was allowed for 3 min to equilibrate, and, again, the frequency scan was performed. Subsequently, these frequency scans were repeated at 24, 35 and 45 °C.

Tube inversion. The sol-to-gel transitions of aqueous acetate-buffered (0.1 M) solutions of F108-OH and F108-Bz at concentrations of 20.3, 26.3 and 33 % w/w were performed by transferring all six samples, prepared in glass vials, into a thermostatically controlled water-bath with a circulator. The temperature was first adjusted to 9 °C, and was subsequently increased gradually up to 30 °C. The samples were allowed to equilibrate for 5 min at each temperature, and were then examined for gel formation by inverting the vial ("tube inversion"). Gel formation temperature was taken as the temperature at which a physical gel was first observed.

Proton nuclear magnetic resonance (^{1}H NMR) spectroscopy. The purity and structure of the F108-Bz amphiphilic block copolymer, its F108-Ms precursor, and the AGE tri-arm star cross-linker, as well as the conversion of the terminal functional groups in each experimental step were determined using ^{1}H NMR spectroscopy. The spectra were recorded in deuterated solvents, in either DMSO-d_6 or $CDCl_3$, using a Bruker 500 MHz Avance spectrometer equipped with an Ultrashield magnet.

Self-healing experiments. The self-healing properties of the F108-based APCNs (33% w/w polymer concentration) were investigated both qualitatively by visual inspection and quantitatively *via* tensile measurements on a mechanical tester (see following paragraph). For both types of experiments, two sample pieces were pressed against each other at their freshly-cut surfaces and held at that position for some time. In the following, we provide the details for the qualitative self-healing procedure. First, one F108-APCN sample dyed (from the stage of its preparation) with methylene blue, and one F108-APCN sample without any dye were cut in half using a razor blade. Subsequently, one piece of the dyed sample and one piece of the non-dyed sample were pressed against each other along their cut surfaces and kept in that position with the aid of a holding apparatus in a closed system. After that, the system was left in the refrigerator for five days before any use.

Tension measurements. The tensile mechanical properties of the F108-APCN were studied using an Instron 5944 mechanical testing apparatus (Instron, Norwood, MA). F108-APCN samples at three different block copolymer concentrations, 20.3, 26.3 and 33.0% w/w, were prepared; moreover, multiple (5-6) samples were prepared for each polymer concentration to afford several repetitions. Furthermore, tension experiments were also carried out on the self-healed F108-APCN samples (see previous paragraph on Self-healing experiments), as well as on the F108-GPEs. The rate of tensile extension was 40 mm min^{-1} for all tension measurements.

Small-angle neutron scattering. Small-angle neutron scattering (SANS) measurements were performed at 25 °C on the D11 instrument at the Institut Laue-Langevin (ILL) in Grenoble, France, covering a total q-range from 0.003 to 0.65 $Å^{-1}$. Samples were measured in quartz cuvettes of 1 mm thickness and data reduction was done using the GRASP software [2], taking into account the detector effectiveness and transmission of the sample. Subsequent SANS measurements were also performed at higher temperatures, upto ~85 °C.

Electrochemical stability measurements. The F108-GPE was sandwiched between a stainless steel disk (15.5 mm diameter, 0.5 mm thickness) and a lithium chip (15 mm diameter). Another stainless steel disk was added as spacer and the whole assembly was sealed in a CR2025 coin cell at 5 tons. Samples were analyzed from open circuit voltage (OCV) up to 0.5 V or 5.5 V *vs.* Li^+/Li at 0.1 mV s^{-1} for linear sweep voltammetry (LSV) or from OCV to 5.5 then 0.5 V *vs.* Li^+/Li at 0.1 mV s^{-1} with 4 scans for CV, all on a BioLogic VMP3 potentiostat.

Ionic conductivity measurements. The ionic conductivity of the F108-GPE systems was characterized using potentiostatic electrochemical impedance spectroscopy (PEIS) measurements. These measurements were conducted by sandwiching the samples between two stainless steel disks, which were sealed between two gold electrodes in a BioLogic Controlled Environment Sample Holder (CESH) under an argon inert atmosphere. Analysis was carried out on a BioLogic VMP3 potentiostat using the following parameters: frequency range from 50 mHz to 1 MHz, Nd: 8 points per decade, and Va = 10.0 mV. The temperature program followed the sequence 80 – 70 – 60 – 50 – 40 – 30 – 20 °C, with a 2 h resting step at each temperature and a temperature accuracy of ± 0.1 °C.

Ionic conductivity measurements under stretching. Two different set-ups were used to test the stretched samples. In "set-up 1", the stretched samples were sandwiched between two stainless steel disks, which were sealed between two gold electrodes in a BioLogic Controlled Environment Sample Holder (CESH) under an argon inert atmosphere. Measurements were carried out as described above at different temperatures. In "set-up 2", the stretched samples were deposited on a copper foil and placed in a setting allowing simultaneous ionic conductivity and membrane thickness measurements (see Figure S10). The whole set-up was placed in a glove box at room temperature. A PalmSens4 impedance analyzer was used for ionic conductivity measurements.

Supporting Figures

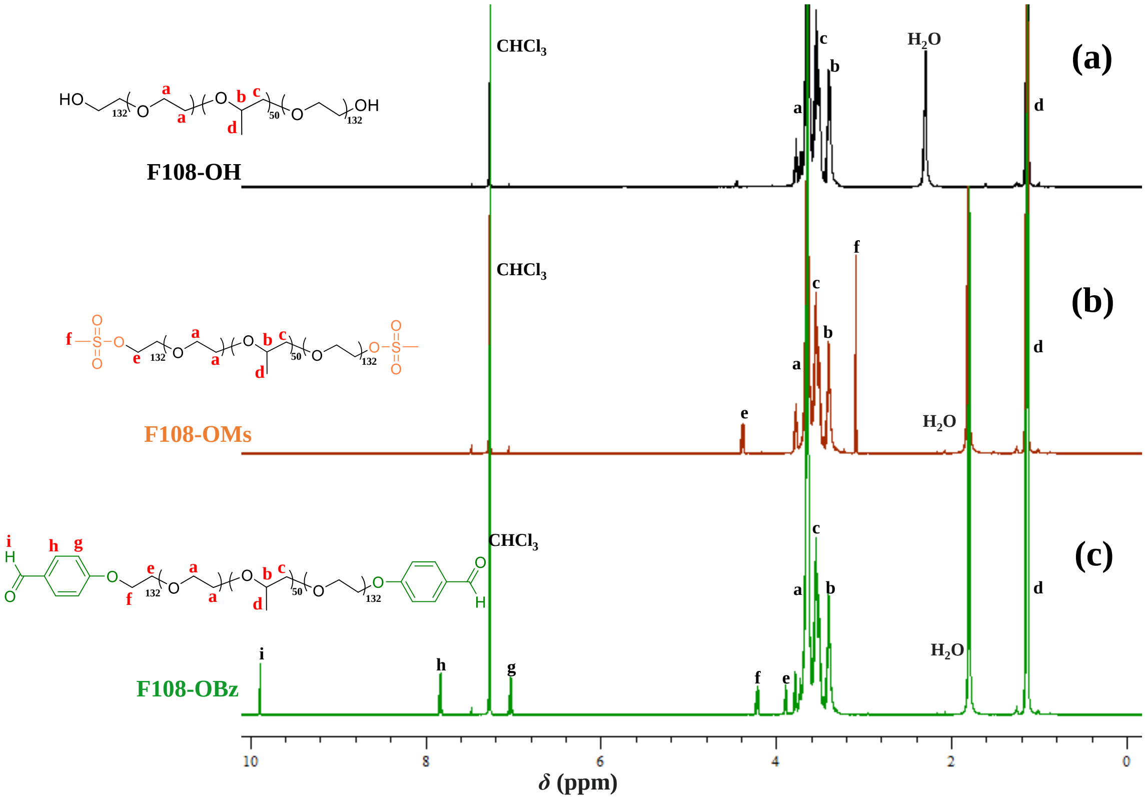

Figure S1. ^{1}H NMR spectra of (a) the starting F108-OH, (b) the intermediate F108-Ms and (c) the final F108-Bz, confirming the complete end-functionalization of the (last) two derivatives.

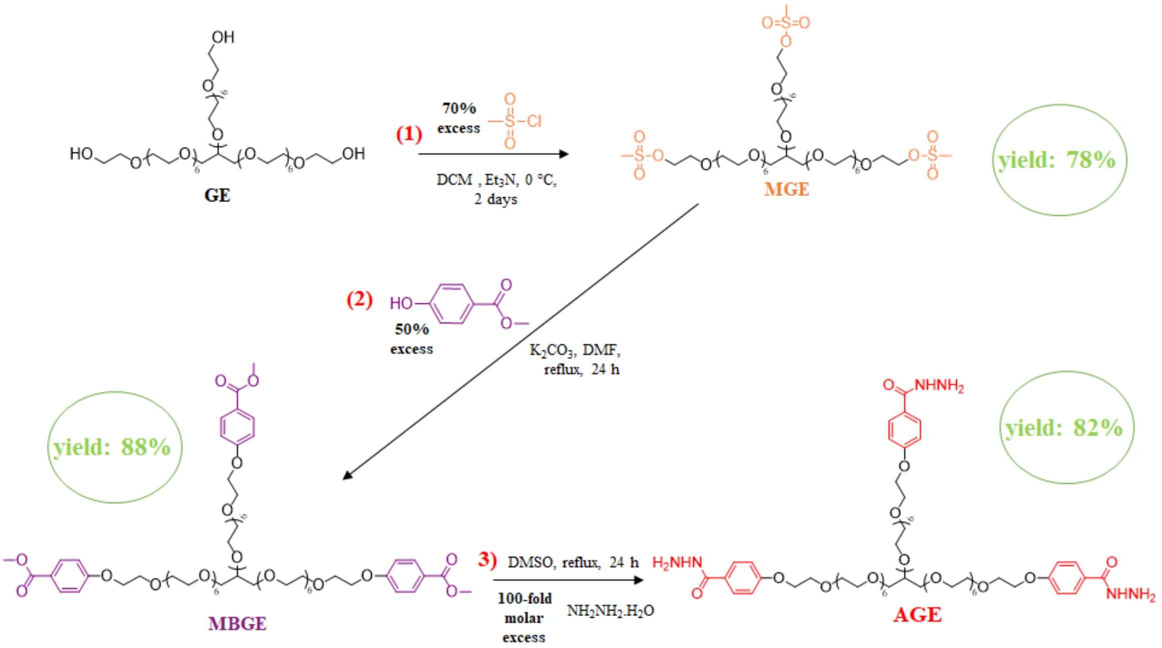

Figure S2. Three-step procedure followed for the attachment of hydrazide groups at the termini of triPEG.

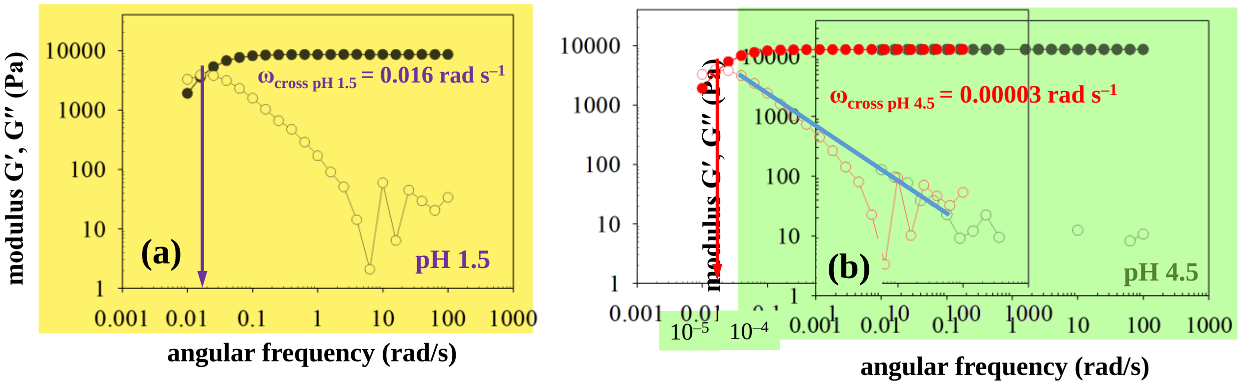

Figure S3. Dependence of the storage modulus, G', and loss modulus, G'', on the angular frequency, ω, at room temperature on a system with the same cross-linking chemistry (homopolymer tetraPEG DYNAgel [3]) as the F108-based APCN system at (a) pH = 1.5 and (b) pH = 4.5. Whereas at pH = 1.5, the two moduli cross within the investigated angular frequency range at ω_{cross} = 0.016 rad s^{-1}, yielding a dynamic bond lifetime of ~60 s, the outside-the-range crossing of the two moduli at pH = 4.5 is determined by superposing [4] the plots at the two pH values, and obtaining an estimation of the value of ω_{cross} = 0.00003 rad s^{-1}, corresponding to a dynamic bond lifetime of ~33 333 s = 9.26 h.

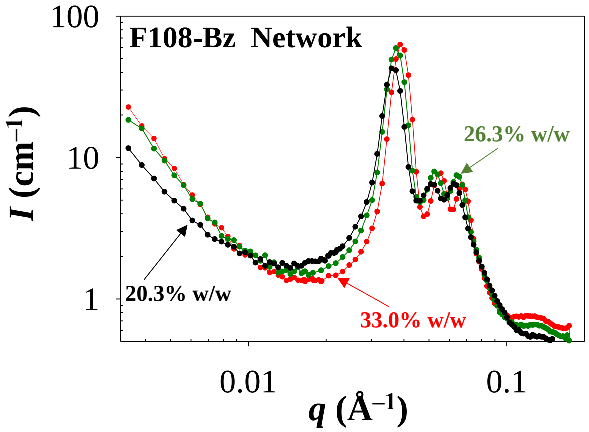

Figure S4. SANS intensity as a function of the magnitude q of the scattering vector for F108-based APCNs in deuterated water at room temperature at three block copolymer concentrations: 20.3, 26.3 and 33% w/w.

Table S1. Results obtained from the SANS profiles (in **Figure S4** above) of three F108-based APCNs in D_2O at room temperature and at three block copolymer concentrations: 20.3, 26.3 and 33.0% w/w.

No.	Polymer Concentration (% w/w)	q_{max} (Å^{-1})	d (nm)	N_{agg}
**s1**	**20.3**	0.0364	24.40	66
**s2**	**26.3**	0.0374	23.74	78
**s3**	**33.0**	0.0391	22.71	86

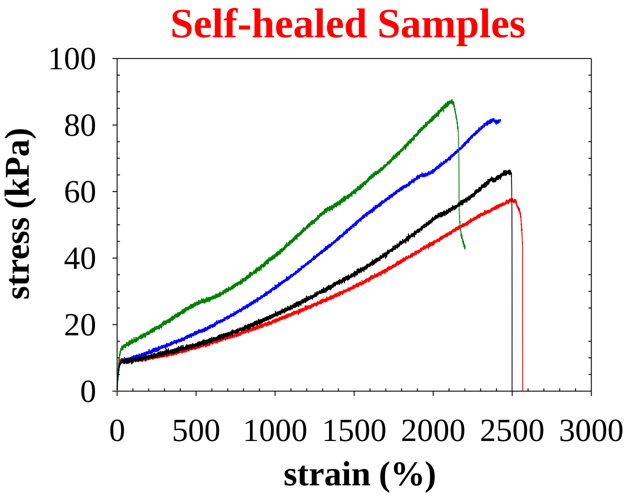

Summary of Results

F108-Bz mdcAPCN	tensile stress at break (kPa)	tensile strain at break (%)
**original**	80.1 ± 11.1	2330 ± 110
**self-healing for 5 days**	74 ± 13	2360 ± 170
**self-healing efficiency (%)**	**93**	**101**

Figure S5. Tensile stress-strain curves of four self-healed F108-APCN samples of 33% w/w polymer concentration, and summary of the calculated values of their self-healing efficiency based both on the tensile stress at break and tensile strain at break.

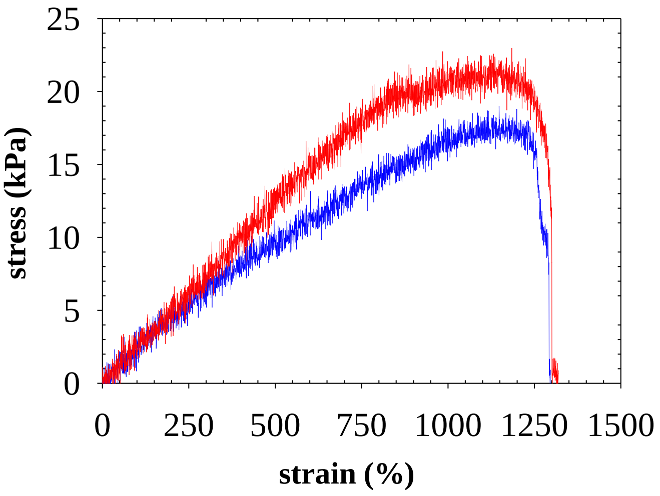

F108.GPE	tensile stress at break (kPa)	tensile strain at break (%)	Elastic modulus (kPa)
	19.6 ± 2.5	1160 ± 91	2.9 ± 0.85

Figure S6. Tensile stress-strain curves of two F108-GPE samples of 33% w/w polymer concentration, and summary of the calculated average mechanical properties, *i.e.*, their tensile elastic modulus and tensile stress and strain at break.

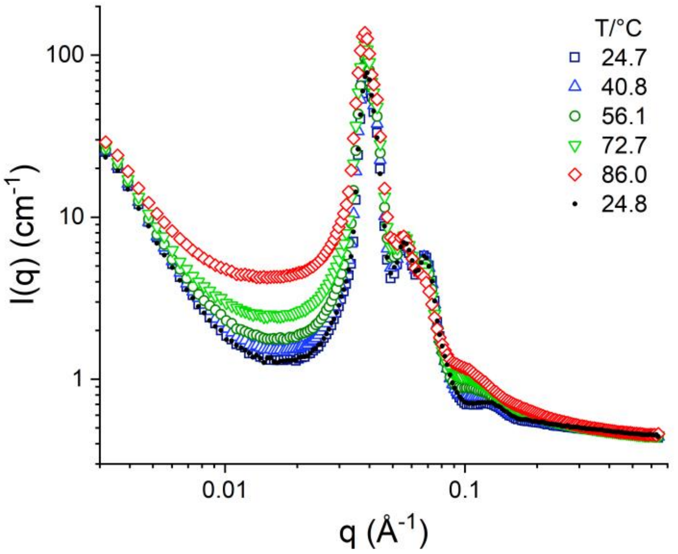

Figure S7. SANS intensity as a function of the magnitude *q* of the scattering vector for the F108-APCN at 33% w/w polymer concentration in deuterated water at temperatures in the range from 24.7 to 86.0 °C, as given in the inset. The (final) run at 24.8 °C was performed after the sample was cooled down from the highest temperature of 86.0 °C.

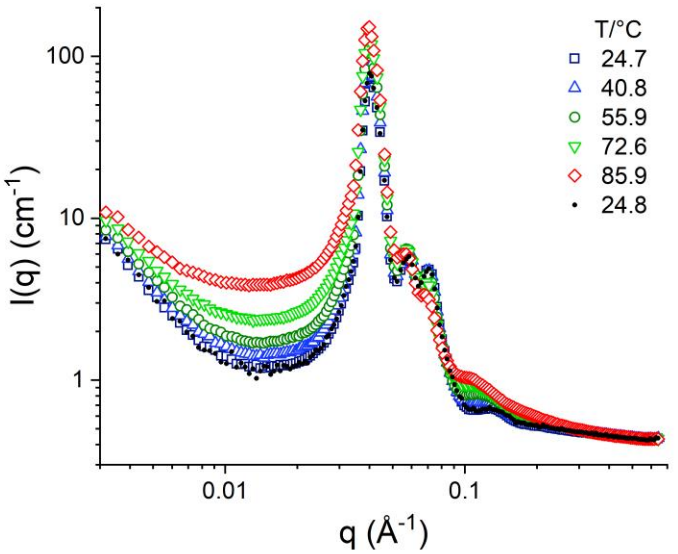

Figure S8. SANS intensity as a function of the magnitude *q* of the scattering vector for a 33% w/w F108-Bz solution in deuterated water at temperatures in the range from 24.7 to 85.9 °C, as given in the inset. The (final) run at 24.8 °C was performed after the sample was cooled down from the highest temperature of 85.9 °C.

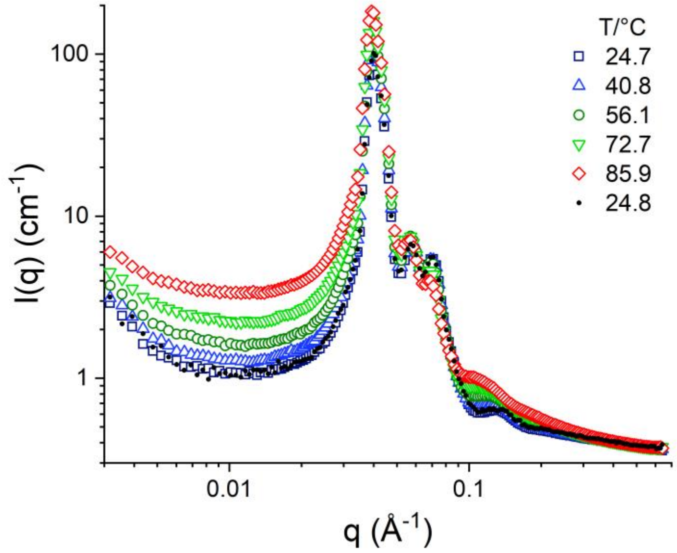

Figure S9. SANS intensity as a function of the magnitude *q* of the scattering vector for a 33% w/w F108-OH solution in deuterated water at temperatures in the range from 24.7 to 85.9 °C, as given in the inset. The (final) run at 24.8 °C was performed after the sample was cooled down from the highest temperature of 85.9 °C.

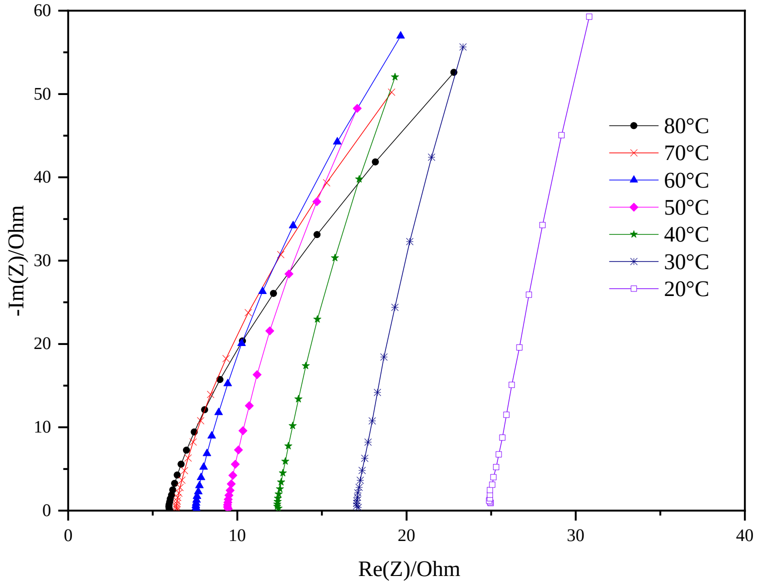

Figure S10. An example of raw data from potentiostatic electrochemical impedance spectroscopy (PEIS) measurements for a 10-fold stretched F108-GPE system obtained at temperatures between 80 and 20 °C, as given in the inset. The calculated ionic conductivities are presented in Figure 11 (red data points). A curved monotonic ("linear") relationship, without semi-circles at high frequencies, was observed in these raw data.

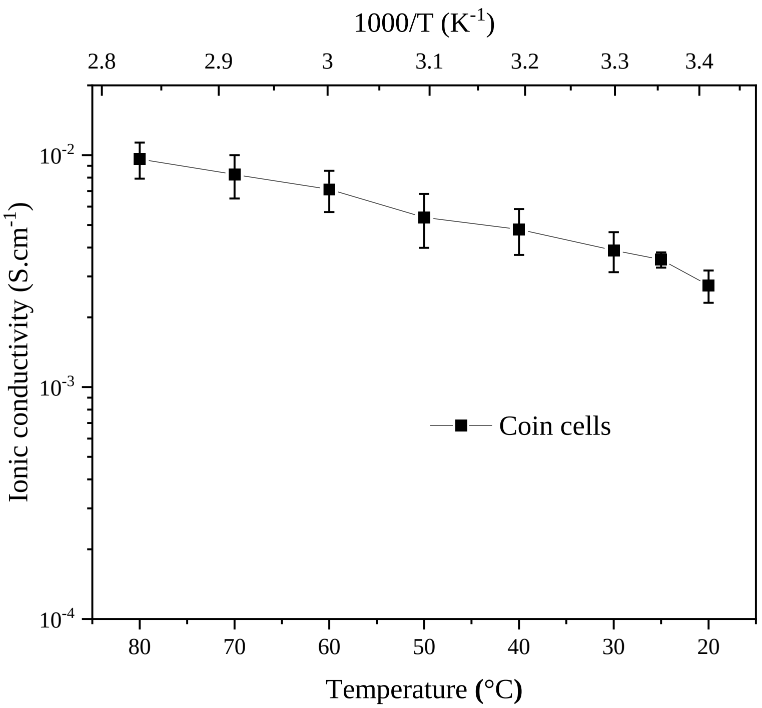

Figure S11. Ionic conductivity repeatability tests ("setup 1") on six different samples of non-stretched GPEs within the temperature range from 80 down to 20 °C. The error bars indicate the variation in the ionic conductivity values.

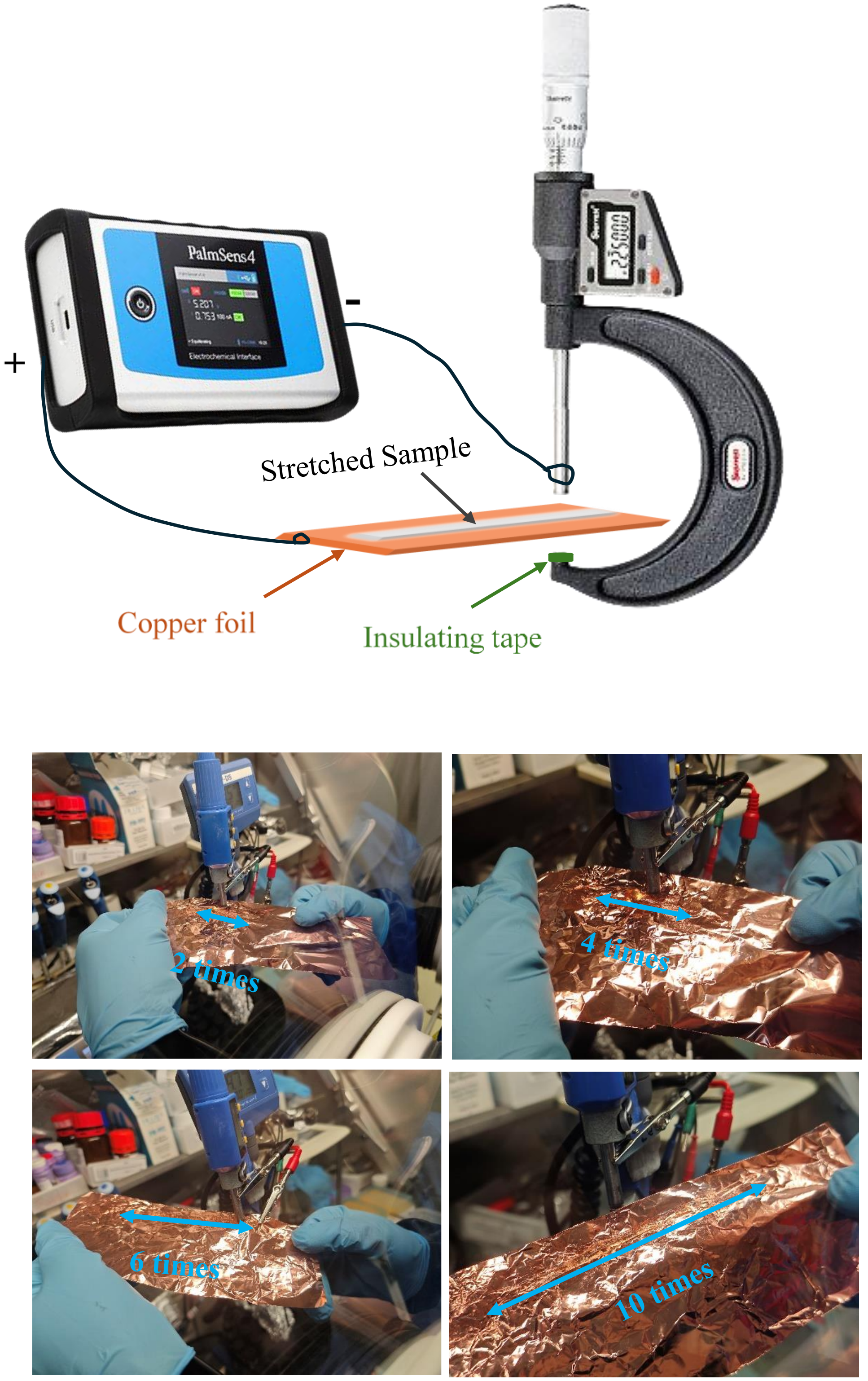

Figure S12. Experimental arrangement ("setup 2") for ionic conductivity measurements under stretching with simultaneous measurement of sample thickness.

References

[1] Apostolides, D. E.; Patrickios, C. S. Model Dynamic Covalent Organogels based on End-linked Three-armed Oligo(ethylene glycol) Star Macromonomers. *J. Polym. Sci.* **2021**, *59*, 2309-2323.

[2] Dewhurst, C. GRASP: Graphical Reduction and Analysis SANS Program for Matlab, 2003.

[3] Apostolides, D. E.; Sakai, T.; Patrickios, C. S. Dynamic Covalent Star Poly(ethylene glycol) Model Hydrogels: A New Platform for Mechanically Robust, Multifunctional Materials. *Macromolecules* **2017**, *50*, 2155-2164.

[4] Deng, G.; Li, F.; Yu, H.; Liu, F.; Liu, C.; Sun, W.; Jiang, H.; Chen, Y. Dynamic Hydrogels with an Environmental Adaptive Self-Healing Ability and Dual Responsive Sol−Gel Transitions. *ACS Macro Lett.* **2012**, *1*, 275-279.
